# A fragile metabolic network adapted for cooperation in the symbiotic bacterium *Buchnera aphidicola*

**DOI:** 10.1186/1752-0509-3-24

**Published:** 2009-02-21

**Authors:** Gavin H Thomas, Jeremy Zucker, Sandy J Macdonald, Anatoly Sorokin, Igor Goryanin, Angela E Douglas

**Affiliations:** 1 Department of Biology, University of York, PO Box 373, York, YO10 5YW, UK; 2 Research Computing, Dana Faber Cancer Institute, 44 Binney Street, Boston, MA 0211, USA; 3 Centre for Intelligent Systems and their Applications, School of Informatics, University of Edinburgh, Appleton Tower, 11 Crichton Street, Edinburgh, EH8 9LE, UK; 4 Department of Entomology, 5136 Comstock Hall, Cornell University, Ithaca, NY 14853, USA

## Abstract

**Background:**

*In silico *analyses provide valuable insight into the biology of obligately intracellular pathogens and symbionts with small genomes. There is a particular opportunity to apply systems-level tools developed for the model bacterium *Escherichia coli *to study the evolution and function of symbiotic bacteria which are metabolically specialised to overproduce specific nutrients for their host and, remarkably, have a gene complement that is a subset of the *E. coli *genome.

**Results:**

We have reconstructed and analysed the metabolic network of the γ-proteobacterium *Buchnera aphidicola *(symbiont of the pea aphid) as a model for using systems-level approaches to discover key traits of symbionts with small genomes. The metabolic network is extremely fragile with > 90% of the reactions essential for viability *in silico*; and it is structured so that the bacterium cannot grow without producing the essential amino acid, histidine, which is released to the insect host. Further, the amount of essential amino acid produced by the bacterium *in silico *can be controlled by host supply of carbon and nitrogen substrates.

**Conclusion:**

This systems-level analysis predicts that the fragility of the bacterial metabolic network renders the symbiotic bacterium intolerant of drastic environmental fluctuations, whilst the coupling of histidine production to growth prevents the bacterium from exploiting host nutrients without reciprocating. These metabolic traits underpin the sustained nutritional contribution of *B. aphidicola *to the host and, together with the impact of host-derived substrates on the profile of nutrients released from the bacteria, point to a dominant role of the host in controlling the symbiosis.

## Background

Obligately intracellular bacteria with very small genomes (< 1 Mb) include important pathogens and required symbionts of parasites and disease vectors [1]. Many are intractable to traditional methods of analysis because they are unculturable and cannot be manipulated genetically. Despite this, informed hypotheses can be constructed from systems-level *in silico *analysis of those bacteria for which full genome sequences are available. In particular, insight into the metabolic capabilities of these bacteria can be obtained from the construction and analysis of metabolic models generated from the inventory of genes with function in metabolism. Of the various methods available, constraints-based modelling using flux balance analysis (FBA) has particular application because it reconstructs flux through metabolism without requiring kinetic or other detailed information on the function of individual metabolic enzymes [2]. Instead, each metabolite is assumed to be in steady-state (i.e. the fluxes producing the metabolite and consuming it are equal), and flux is optimised to a desired output, also known as the objective function, usually biomass production. The purpose of this study was to reconstruct and analyse the metabolic network of an unculturable obligately symbiotic bacterium and, from this, deduce how the bacterium may be controlled by its host. We focused on the bacterium *Buchnera aphidicola *APS from the pea aphid, which has a 0.64 Mb genome [3]. *B. aphidicola *provide aphids with essential amino acids (EAAs), nutrients which the insect cannot synthesise *de novo *and which are in short supply in the diet of plant phloem sap [4]. Remarkably, the gene content of *B. aphidicola *is a subset of the *E. coli *K-12 genome [3,5], allowing nearly all *Buchnera *gene products to be assigned confident functional assignments. In this way genomic and systems biology tools developed for *E. coli *can be used to explore the metabolic properties of this symbiotic bacterium [6]. Constraints-based modelling using FBA has been applied to a variety of organisms, from *E. coli *to humans [7,8] and various symbiotic bacteria [9,10] including *B. aphidicola*. In this study, we have created a high quality manually constructed metabolic model for *B. aphidicola *that is more biologically realistic than previous studies [e.g. [1,9,10]]. In particular, we have imposed the requirements that, first, the cell synthesises the cofactors needed by other enzymes that operate in the network; and, second, EAAs are exported at empirically determined rates. This biologically realistic model provides the basis to assess the genetic robustness of the metabolic network and explore how the sustained release of EAAs is shaped by the structure of the metabolic network and nutrient supply from the insect host.

## Results and discussion

### The metabolic network of *Buchnera aphidicola *APS

**Figure 1:**
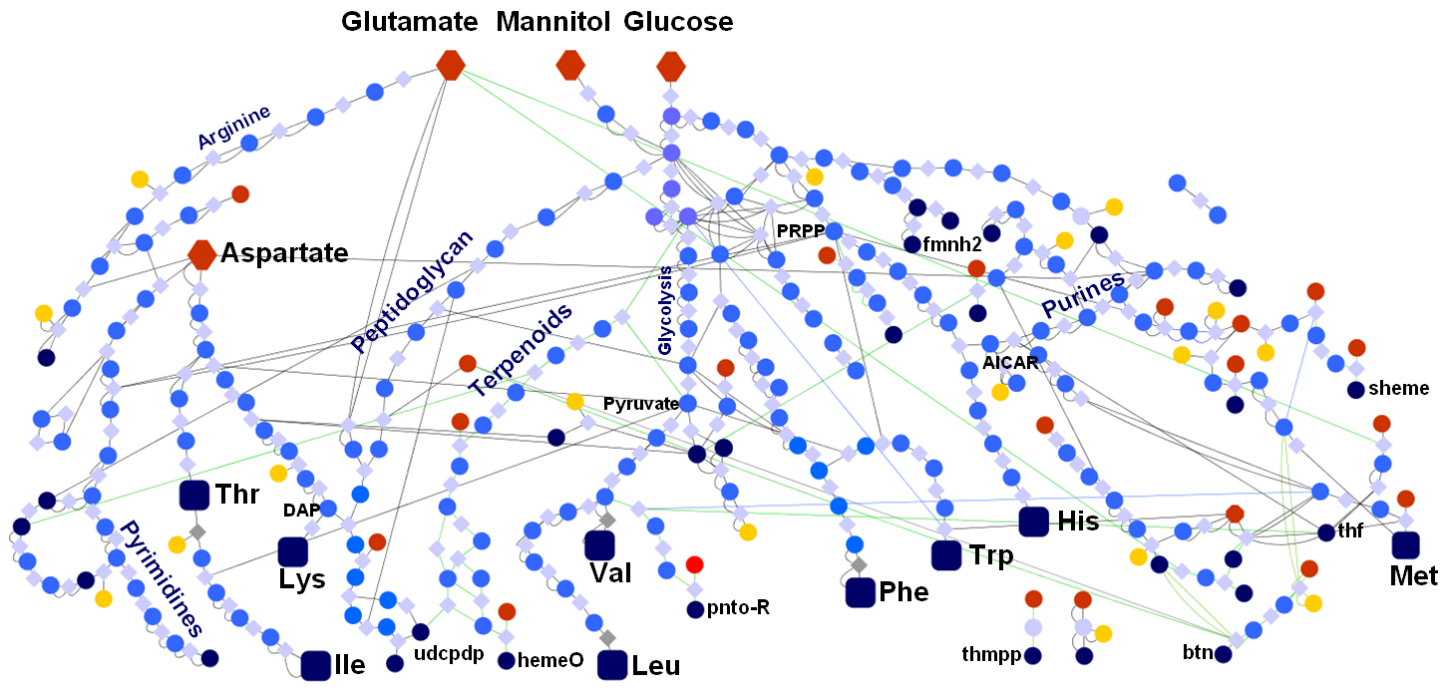
**Schematic layout of the metabolic pathways of *Buchnera aphidicola *APS illustrating the carbon flow from the main precursors to EAAs (larger symbols)**. Metabolites consumed in the model are coloured red, by-products are yellow, and components of the biomass reaction are blue.

The metabolic scope of the network (iGT196, see Materials and Methods) is small, comprising 196 gene products, 240 compounds and 263 reactions, only 39% of the compounds and 27% of the reactions in the *E. coli *iJR904 model. The limited number of metabolic pathways (Fig. 1) includes central metabolism and biosynthetic routes for nucleotides, amino acids and cofactors. Notably 35% of all reactions in the network are involved in EAA biosynthesis.

The metabolic network of APS is poorly connected in comparison to *E. coli*. In the reaction graph, the modal path length between every pair of compounds, the maximal path length and %-unreachable nodes are all higher for APS than for *E. coli *(Table 1). Also, the node distribution for both the compound graph and the reaction graph in both *E. coli *and APS had no significant match to the power law, as determined by the minimal likelihood method (Table 1). This is consistent with recent work [9] demonstrating that the scale-free distribution reported earlier for *E. coli *arose from inappropriate combining of frequencies for different nodal values.

**Table 1:** Topological parameters for the metabolic networks of *E. coli *and *Buchnera *APS

Bacterium (model)	Reaction graph			Goodness of fit of node distribution^2^					
	
	Path length^1^			Compound graph			Reaction graph		
	Mode	Max. path length	No. of unreachable nodes/total no. of nodes (%)	In-degree	Out-degree	Total	In-degree	Out-degree	Total

*Buchnera *iGT196	8	25	19,493/55,932 (34%)	1.622 (0.448)	2.020 (0.211)	1.700 (0.487)	2.411 (0.115)	2.327 (0.114)	1.701 (0.344)
*E. coli *iJR904	6	19	108,467/369,056 (29%)	1.911 (0.222)	1.905 (0.212)	1.622 (0.448)	2.104 (0.127)	2.114 (0.112)	1.623 (0.333)

^1 ^number of reactions between every pair of metabolites.

^2 ^In-degree is the number of substrates and out-degree is the number of products for the reaction graph. The value of gamma is shown with the Kolmogorov-Smirnov statistic in parentheses, with critical values (α = 0.01) of 0.050 for *E. coli *and 0.110 for *Buchnera*.

### Analysis of the metabolic model (iGT196) using Flux Balance Analysis

We applied FBA to explore the distribution of metabolite flux across the network, optimised to maximise biomass production. Two novel features were included in our model to promote biological realism. The first, which we term 'cofactor constraints', is the requirement for the cell to synthesise at low levels all the cofactors needed by other enzymes in the network, if it has the pathways available for making these compounds (see Fig. 1 and Additional File 1). This requirement has not been included in multiple previous studies of *E. coli *metabolic networks, including the published analysis of the iJR904 model [7], resulting in many pathways essential for *in vivo *viability of *E. coli *having zero flux *in silico*. The second modification to the model was the requirement that EAAs are exported. An estimate of EAA export was obtained empirically using the pea aphid-*Buchnera *symbiosis reared on chemically-defined diets. It varied among the amino acids, from 22% (histidine and tryptophan) to 50% (threonine) of the amount synthesised (see Additional File 2). The FBA model is, consequently, representative of a functional symbiotic bacterium; it is called the 5.21 model (V_growth _is 5.21, see Additional File 3). For FBA analysis, all reactions in the network were permitted to be active simultaneously. This condition is realistic because *B. aphidicola *lacks almost completely transcriptional regulation [10,11] and sequence analysis of enzymes subject to feedback inhibition in *E. coli *suggests these features have been lost from *B. aphidicola *[12].

### Genetic robustness of the metabolic network for *in silico *growth

**Figure 2:**
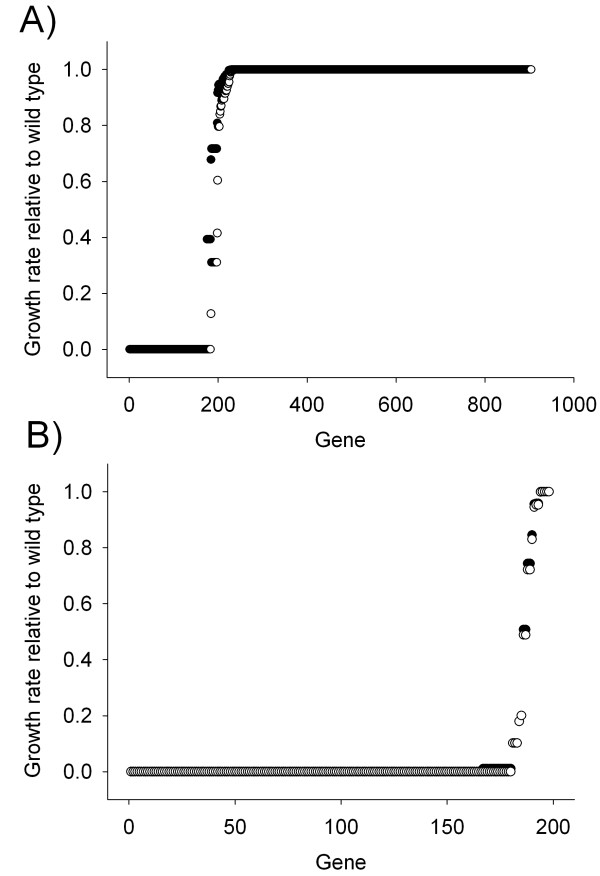
**Genetic robustness of A) the iJR904 metabolic network for *E. coli *K-12 and B) the iGT196 metabolic network of *B. aphidicola***. Data are presented from single deletion experiments simulated using FBA (●) and linearMOMA (○) in the COBRA software.

The genetic robustness of iGT196 was explored by removing single genes from the *in silico *organism, thereby setting zero fluxes through any reactions for which that particular gene product is essential. The iJR904 model of *E. coli *K-12 provided a related robust network for comparison [7]. When deletion of a particular gene resulted in a > 99% decrease in the growth flux it was considered essential, and remarkably this was true for 84% of the genes in iGT196, while the equivalent value for iJR904 was 19%. This significant difference in essentiality is illustrated graphically in Fig. 2, which plots the relationships between the genes in the models and the % of the original biomass production remaining when they have been removed and demonstrates that the APS metabolic network is extremely fragile. The severely limited redundancy in the APS network included the ability to utilise each of two alternative exogenous carbon sources (glucose and mannitol) and either the *hpt *or *gpt *gene product as isoenzymes for the guanine phosphoribosyltransferase reaction (see see Additional File 4 for details). The latter is the only example of true biochemical redundancy in the network. The *in silico *organism is able to grow at a very low rate in the absence of the *nuo *genes indicating that the lack of a functioning membrane-bound NADH dehydrogenase, and hence of a functional respiratory chain, can be tolerated by a network relying on substrate-level phosphorylation. In contrast, the terminal reductase of the respiratory chain, the cytochrome *bo *oxidase, cannot be eliminated because reduced quinone is required for the dihydoorotic acid dehydrogenase reaction in pyrimidine biosynthesis.

We also analysed single gene deletions by the linear minimisation of metabolic adjustment (linearMOMA) method [13]. This analysis produced the same set of genes as in Fig. 2 and Additional File 4 apart from a requirement for *nuo *genes (the large changes in the overall pathways fluxes resulting from deletion of *nuo *genes were not tolerated by linearMOMA (Additional File 4)). Hence, 184 of 196 (~94%) genes in the linearMOMA model for iGT196 are essential for growth, compared to 183 of 904 (~20%) for iJR904; 47 (26%) of the required genes were shared between *B. aphidicola *(iGT196) and *E. coli *K-12 (iJR904).

These data demonstrate that APS does not conform to the generality that metabolic networks are complex and robust [14], and suggest that the properties of *B. aphidicola *are close to those of the postulated minimal metabolic network [15]. The fragility of the metabolic network suggests that *B. aphidicola *is intolerant, first, of mutations that eliminate metabolic reactions unless that loss is compensated for by enhanced metabolic support from the host, and, second, of drastic changes in conditions, suggesting that the environmental conditions in the symbiosis may be relatively uniform. The condition of *B. aphidicola *in the symbiosis is consistent with this interpretation. The bacterial cells are restricted to a single insect cell type, the bacteriocyte, the sole function of which appears to be to house and maintain the bacteria; the cytoplasm of the bacteriocyte is packed with bacterial cells, each of which is enclosed by an insect membrane, known as the symbiosomal membrane [4]. The metabolic traits of the bacteriocyte and transport properties of the symbiosomal membrane are predicted to be adapted to support and control the fragile network of these symbiotic bacteria by regulating metabolic flux to the bacteria and the physicochemical conditions experienced by each bacterial cell.

### Metabolic adaptation of APS for the symbiotic function of EAA production

**Figure 3:**
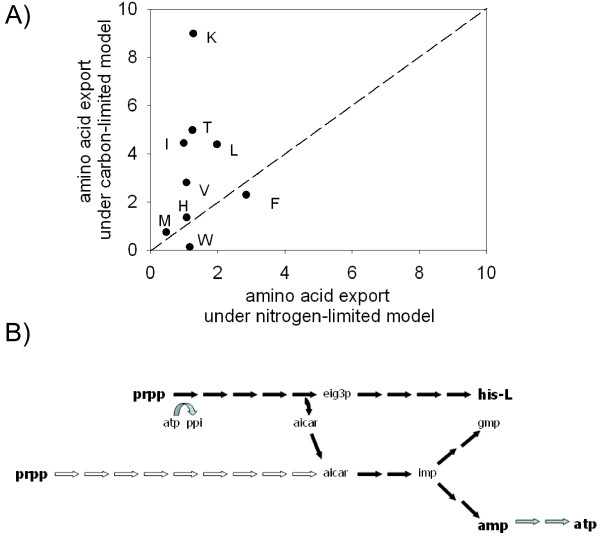
**EAA production by *Buchnera aphidicola *APS**. (A) export under different substrate inputs. Equal export under the two models is shown by the dashed line. Standard single letter abbreviations are used for EAAs. (B) Metabolic coupling between the purine and histidine biosynthetic pathways. The reactions present in *E. coli *are represented with white arrows representing reactions absent from *B. aphidicola *and grey arrows indicating the recycling of ATP between the purine and histidine biosynthetic pathways. Full metabolite names can be found in Supplementary File 1.

Host demand for EAAs derived from *B. aphidicola *is predicted to vary because the amino acid composition of the aphid diet of plant phloem sap is influenced strongly by environmental conditions and plant age and species [4]. Several lines of evidence suggest that the amount of EAAs released varies with host demand but it is not understood how the host communicates its nutritional demand to *B. aphidicola*. One hypothesis is that EAA production is controlled by the supply of carbon and nitrogen substrates from the host. In iGT196, the principal carbon sources are glucose and mannitol, and the nitrogen sources are the amino acids aspartate, glutamate and glutamine. To test this hypothesis, carbon-limited and nitrogen-limited models were generated by increasing supply of either nitrogen or carbon sources, respectively. Consistent with prediction, these changes to the inputs to the APS network had substantial impacts on the output of EAAs (Fig. 3A), demonstrating that host regulation of symbiotic function by nutrient supply is metabolically feasible. EAA production by APS is, thus, poised to vary with substrate availability from the host.

An extension of the hypothesis above is that the profile of EAAs released from APS is responsive to the nutritional inputs from the host cell. These computed flux changes are unlikely to be dampened or prevented *in vivo *because the APS network has very little redundancy and is not subject to transcriptional or post-translational controls [10-12]. It is known that the EAA production by *B. aphidicola *varies with host demand, as dictated primarily by dietary supply of amino acids [4]. The *in silico *analysis reported here suggests that the supply of key precursors across the symbiosomal membrane may be sufficient to regulate EAA export from the bacteria. Ongoing research is exploring how the individual carbon and nitrogen sources influence the pattern of flux through the different EAA biosynthetic pathways. Intra- and inter-specific variation in aphid dietary requirements for EAAs has been reported [4,16]. Although some of this variation may be attributed to the gene complement of different strains of *B. aphidicola*, the results in Fig. 3A raise the possibility that the differences in the metabolic and transport properties of the host bacteriocyte may also play a key role in determining the EAA profile released from *B. aphidicola *in different aphids.

### Truncation of the purine biosynthesis pathway in the APS network couples bacterial growth to EAA production

Visual inspection of the APS metabolic network revealed a particularly interesting feature, namely the proximal truncation of the purine biosynthesis pathway (Fig. 1 and 3B). In *E. coli *and many other bacteria, the histidine and purine biosynthesis pathways are linked via an intermediate, AICAR, which recycles the backbone of the ATP molecule used in a proximal reaction of the histidine biosynthetic pathway (Fig. 1 and 3B). Both pathways start with PRPP and in *E. coli *flux through the purine pathway is 3–4 times greater than through the histidine pathway [17]. In APS, AICAR (and not PRPP) is the precursor for purine synthesis (Fig. 3B), and generation of AICAR at sufficient rates to meet the purine requirements of *B. aphidicola *depends on high flux from PRPP through the histidine biosynthesis pathway. The truncated AICAR-dependent purine biosynthesis is also found in *Blochmannia*, a related intracellular bacterium that may also overproduce EAAs [1], but a cross-genome analysis of purine biosynthesis genes performed using PhydBac [18] revealed no other bacteria with this truncation. This apparently unique coupling predicts that production of the EAA histidine is essential for purine biosynthesis. The absolute connection between the two pathways was confirmed by examining the flux correlations of both pathways (see Additional File 5).

To assess whether we could model the evolution of the reduced *Buchnera *network with this key interdependence, we adopted an established procedure using *E. coli *iJR904 as an approximation to the ancestral non-symbiotic metabolic network [19] and applied a sequential gene deletion method to evolve organisms with the same metabolic outputs as iGT196. A total of 214 genes were retained in all of the 500 simulations performed, comprising 148 (76%) of the 196 genes in iGT196 and a further 66 genes absent from iGT196 (Additional File 6). Neither this study nor Pal *et al*. (2006) could replicate the proximal truncation of the purine biosynthetic pathway (Fig. 3B). Whilst the greedy algorithm and sampling numbers used by Pal *et al*., (2006) and herein potentially limit the full interpretation of this experiment, it is clear that, unlike the evolutionary loss of various other metabolic genes, the truncation of the purine pathway was an improbable evolutionary step. To our knowledge, this type of host-symbiont linkage has not been described previously for any symbiosis. There is, however, evidence for coupling of the metabolism of nitrogen-fixing rhizobia and the surrounding plant cells in legume root nodules, such that rhizobial access to host amino acids is dependent on the release of the nitrogen-fixation product, ammonia [20]. Further research on the interface between the metabolic networks of *B. aphidicola *and its host may reveal similar couplings between nutrient supply to *B. aphidicola *and bacterial overproduction of EAAs. Such couplings would preclude the evolution of bacteria with reduced export of EAAs, despite the evidence that EAA export is a costly trait for *B. aphidicola *(when FBA is repeated in conditions where EAA export was removed, biomass production increased by 7% from 5.21 to 5.58 units). The metabolic coupling is in the selective interest of the *B. aphidicola*. Because *B. aphidicola *is an obligately vertically transmitted microbe with a small effective population size, any short-term growth advantage of reduced EAA export to the host would rapidly translate into depressed fitness of both the host and its bacterial complement.

## Conclusion

Systems level *in silico *analysis has shed light on the evolution and function of a symbiotic bacterium with a small genome. From the properties of the reconstructed metabolic network of *B. aphidicola*, adaptations for the symbiotic lifestyle can be identified. The fragile metabolic network suggests that the symbiotic environment is benign and not subject to drastic fluctuations, and host controls over bacterial metabolism are indicated by the responsiveness of the essential amino acid profile released from the bacteria to the host supply of carbon and nitrogen substrates. The coupling of purine synthesis (and hence sustained bacterial growth) to the overproduction of histidine transferred to the host is potentially one route by which this bacterium is bound to the cooperative lifestyle. Systems level analysis of other taxa will establish the generality of these key traits of metabolic fragility, flexibility and coupling among symbiotic bacteria with reduced genomes.

## Methods

### Description of the iGT196 model

The iGT196 model comprised a subset of *E. coli *K-12 iJR904 model [7] derived by eliminating all reactions coded by genes without homology in APS or present in APS but either not connected to the biomass reaction (*apaH*, *gloB*, *lig*, *gltX*, *suhB*, *mutT*) or representing isolated enzymes in missing pathways (*serC*, *fabB*, *fabG*, *fabI*, *gltX *and *hemC*). Other reactions supported by experimental evidence were added (indicated by 'inferred' in Additional File 3). These are transaminase reactions (VALTA, PHETA1, LEUTAi, ILETA) assumed to be present in *B. aphidicola *because the amino acids that these reactions are involved in synthesising are made in *B. aphidicola APS*; the initial step in the isoleucine biosynthesis pathway (THRD_L), which has been detected in isolated extracts of *B. aphidicola *APS (Douglas, A.E, unpublished results); the true 'orphan' (PMDPHT, for which no encoding gene is known in *E. coli*); and one spontaneous reaction (HCO3E). Pathways and reactions were checked using the EcoCyc Pathway/Genome database [21]. Mapping of genes used reciprocal BLAST searches to identify orthologues present in both bacteria, the results of which are available in *Buchnera*BASE [6]. The model has been submitted in SBML to the BioModels database  with accession number MODEL7434234848. The reconstruction is also available in *Buchnera*Cyc  a BioCyc genome/pathway database created and adopted for this project using the Pathologic software [22]. The reactions and metabolites present in iGT196 are provided in Additional File 3.

The biomass reaction for the model was derived from that used in iJR904 which was based on experimental data for *E. coli *K-12[7]. The coefficients for EAAs were modified to include empirical data for EAA export from *B. aphidicola *to the aphid (Additional File 2) and the ratios of the four different deoxynucleotide triphosphates required for growth were changed to reflect the GC content of the *Buchnera *genome (26.4%) compared to *E. coli *K-12 (50.8%).

The flux balance analysis (FBA) was performed using the Fluxor toolkit, which uses linear optimization algorithms provided by the GLPK and OOQP toolkits. The Fluxor software is freeware available at . The 5.21 model provides glucose and mannitol as carbon sources and glutamine, glutamate and aspartate as nitrogen sources and precursors for biosynthesis (Additional File 3). The insect cells bearing *Buchnera *are well-tracheated indicating that oxygen supply to *Buchnera *is not a constraint and hence the model operates using an aerobic respiratory chain. Identical data were also obtained by analysing the model using the COBRA software [13].

### Analysis of network properties

The metabolic network of *Buchnera *was represented as a directed bipartite graph *G *= (*C, R, L*), where *C *is a set of nodes representing compounds (metabolites), *R *is a set of nodes representing reactions and *L *is a set of directed links, i.e. an ordered pair of nodes from one *C *to one *R*. Compound *c*_*i *_is involved in reaction *r*_*j *_if and only if there is link between node *c*_*i *_and *r*_*j*_. This directed graph is referred to as the "reaction graph". The reaction graph can also be simplified by removing all reaction nodes and replacing them with links connecting each substrate with all its products [23,24]. These reduced graphs are referred as "compound graphs" as only the compound nodes remain. We created reaction graphs without taking into account subcellular localisation and diffusion and transport reactions. For the analysis of the network properties, we have removed the currency metabolites H2O, ATP, NADH, NAD, NADPH, NADP, O2, ADP, PI, COA, CO2, PPI, NH4, AMP and H. We used a maximum likelihood approach [25] to fit the distribution of both the in- and out-degree to a power law. Analysis was performed using a Java application based upon the JUNG graph Java library  and is available from the authors asorokin@inf.ed.ac.uk upon request. The network diameter is the maximal length of the shortest path between two reachable nodes in the network. Calculation of the shortest path between two compounds using the reaction graph considers the two links that join two compounds to a reaction as a single link. We used a maximum likelihood approach [25] to fit the distribution of both the in- and out-degree to a power law for both iJR904 and iGT196. This approach differs from a recent analysis [24] in which the data were binned with the first three bins combined before fitting to power law by least squares using a log/log plot. For each pair of compound nodes in the reaction graph shortest path distance was calculated by breadth-first search. Where no path existed between two nodes, the pair was marked as unreachable.

### Fragility analysis

For the fragility analysis the 5.21 FBA model for iGT196 was used as the starting point. Using FBA, we simulated the consequences of making individual reactions within the model have zero flux and recorded the V_growth _of the resulting mutant (Additional File 4). We investigated a variety of different cut-offs for essentiality, ranging from 1% to 99% which did not give very different results (97 to 84% essential for iGT196 and 23 to 19% essential for iJR904). For the iJR904 mode we simulated aerobic growth on a glucose minimal medium. We also performed a similar single gene deletion analysis of the iGT196 model and of the iJR904 model using the COBRA toolbox FBA software as described previously [13]. Two methods were used for calculation of resultant relative growth rates: standard FBA and linearMOMA [13].

### Impact of nutrient inputs on EAA release

The two models used were: carbon-limited (upper limits for uptake of amino acids aspartate, glutamate and glutamine at 500, and carbon sources glucose and mannitol at 100 and 50, respectively) and nitrogen-limited (upper limit for amino acid uptake at 100, and glucose and mannitol uptake at 500 and 250, respectively). Uptake of nitrogen sources in the nitrogen-limited model and of carbon sources in the carbon-limited model were maximal.

### Evolution of minimal metabolic networks

The evolution of minimal networks were simulated by the methods of Pal *et al*., (2006) with the biomass reactions from iGT196 and iJR904, both with riboflavin included in the biomass reaction (as in Pal *et al*., (2006)). Initial FBA analysis using iJR904 modified to have the biomass reaction from iGT196 did not produce a feasible solution because the biosynthetic pathways to biotin and thiamine diphosphate were incomplete. Both of these cofactors were added to iGT196 as 'cofactor constraints' and were absent from the original iJR904 biomass reaction which lacked these additional criteria for growth. Therefore we added an efflux reaction for S-adenosyl-4-methylthio-2-oxobutanoate (amob) and for 4-hydroxy-benzyl alcohol (4 hba) which are produced but not consumed in the biotin and thiamine diphosphate biosynthesis pathways, respectively, and also an uptake reaction for pimeloyl-CoA (pmcoa) which is consumed but not produced during biotin biosynthesis. The analysis was performed as described previously [19] using the Fluxor software running on Orchestra (a 100-node cluster) and was repeated 500 times.

## Authors' contributions

The construction and analysis of the model was performed by GHT, JZ, SJM, AS and IG. AED, GHT and JZ conceived the study and designed and coordinated it. All authors contributed towards the writing of the manuscript.

*Data deposition *The model is available in BioModels database (MODEL7434234848), and the reconstruction is available at . The authors declare no competing financial interests. Correspondence and requests for materials should be addressed to GHT.

## Supplementary Material

Additional file 1 **Additional Methods.** This file contains additional methods for the construction and validation of the model and the flux correlation method with references. Click here for file

Additional file 2 **Quantification of essential amino acid release from *B. aphidicola *APS.** These data demonstrate the contribution of *B. aphidicola *APS-derived essential amino acids to protein growth of 2-to-7-day-old larval pea aphids (clone LL01) on chemically-defined diets. Click here for file

Additional file 3 **Description of the iGH196 model and fluxes from the 5.21 model.** The Excel sheet describes the gene/reaction relationships in the model and the fluxes in the 5.21 model. Click here for file

Additional file 4 **Analysis of the FBA simulations for non-essential genes.** The file summarises the results from our analysis of the FBA simulations for non-essential genes from iGT196 by our own method and also by using the linearMOMA method. Click here for file

Additional file 5 **Flux correlation of the histidine and purine biosynthesis pathways.** Pairwise scatterplots showing correlation of fluxes in reactions of the histidine and purine biosynthetic pathways of *Buchnera aphidicola*. Click here for file

Additional file 6 **List of APS genes and the frequency of their retention in evolved symbionts.** This file lists the APS genes and the frequency of their retention in evolved symbionts from Pal et al,. (2006) and this study. Click here for file
