# Supplementary material for: A fragile metabolic network adapted for cooperation in the symbiotic bacterium Buchnera aphidicola
Source: BMC Syst Biol. 2009 Feb 21;3:24. doi: 10.1186/1752-0509-3-24 (PMC2649895; doi:10.1186/1752-0509-3-24)
Supplement: Additional file 1 — Additional Methods. This file contains additional methods for the construction and validation of the model and the flux correlation method with references. [file 1752-0509-3-24-S1.doc]

A fragile metabolic network adapted for cooperation in the symbiotic bacterium *Buchnera aphidicola*

Gavin H. Thomas^1*^, Jeremy Zucker^2*^, Sandy J Macdonald^1^, Anatoly Sorokin^3^, Igor Goryanin^3^ and Angela E. Douglas^1#^

Additional File 1 Methods

**Additional details of the construction and validation of the metabolic model, iGT196**

The metabolic model was built in stages based on comparisons to *E. coli* and was described using the *E. coli* iJR904 element and charge-balanced metabolic model(Ree*d et a*l., 2003) with a number of small naming differences introduced from a more current model (Adam Feist, personal communication). A number of pathways and reactions were also checked using the EcoCyc Pathway/Genome database(Kesele*r et a*l., 2005).

The genes encoding several predicted metabolic reactions were not included in iGT196, either because they were not connected to the biomass reaction (ApaH, GloB, Lig, GltX, SuhB) or because they encoded isolated enzymes in missing pathways (SerC and FabB). Our analysis revealed that *B. aphidicola* APS contains all the genes required to encode a pathway for the biosynthesis of UDP-N-acetyl-muramate from D-fructose-6-P and the subsequent conversion of this to make peptidoglycan. This was not evident from the previous reconstruction *of* *B. aphidicola* APS(Zientz, Dandekar, and Gross, 2004). The specific uptake reaction for pantetheine 4'-phosphate (UP_pan4p) is included in iGT196, to support the last two reactions (coded by genes in APS) for *de novo* coenzymeA biosynthesis.

Two reactions absent from iJR904 are included in iGT196. (1) the reaction required to synthesise FMNH_2_, a cofactor for the AroC enzyme, is catalysed by the CysJ protein, (present in *B. aphidicola* APS); the code FMNREDUCT is assigned to this reaction. (2) the reaction for the synthesis of acyl carrier protein (ACP) from apoACP by the AcpS protein, assigned the code ACPS1. ACP is a biomass component. The protein substrate was set up as an influx reaction (which has been labelled an EN_ reaction to distinguish this from a normal uptake reaction). The properties of FMNREDUCT and ACPS1 are taken from Ecocyc (Kesele*r et a*l., 2005).

The cofactors added to the biomass reaction as our ‘cofactor constraints’ that were not present in the iJR904 biomass reaction (and hence had no flux in these FBA simulations) were (R)-pantothenate (pnto-R), sirohaem (sheme), thiamine diphosphate (thmpp), biotin (btn), heme O (hemeO), undecaprenyl diphosphate (udcpdp) and tetrahydrofolate (thf). We also required reduced glutathione (gthrd) to be produced to maintain the redox state of the cell. All of these additional components were given arbitrary small coefficients in the biomass reaction (0.00005), such that the model has to be able to make these compounds, but only in relatively small amounts.

In the final model of metabolism, a number of compounds were produced as dead-end metabolites: adenine (ade) and 5-methylribose (5mtr), both produced during spermidine biosynthesis; S-ribosylhomocysteine (rhcys) produced during the recycling of S-adenosylhomocysteine; succinate (suc) produced during lysine biosynthesis and glycerol (glyc) produced during cardiolipin biosynthesis. These compounds might be recycled by uncharacterised enzymes in *B. aphidicola* APS, but in the absence of any experimental data, they were removed from the cell via efflux reactions.

**Flux correlation of histidine/purine synthesis pathways.**

Metabolic coupling of these pathways was investigated by performing a flux correlation analysis of reactions within these pathways. The COBRA toolbox FBA software was used for this analysis and the method is described in detail in Becker *et al* (Becke*r et a*l., 2007). Briefly, pairwise scatterplots were calculated for all possible combinations of reactions within the given pathways, allowing the relationship, or flux correlation, between each pair of reactions to be determined. Positive correlation between a pair reactions was inferred if the majority of points in a given scatterplot fell on a diagonal line with a positive gradient. Conversely, if the majority of points on a scatterplot fell on a diagonal line with negative gradient then a negative correlation was inferred. A random scattering of points indicated zero correlation.

Additional Methods reference List

Becker,S.A., Feist,A.M., Mo,M.L., Hannum,G., Palsson,B.O., and Herrgard,M.J. (2007) Quantitative prediction of cellular metabolism with constraint-based models: the COBRA Toolbox *Nat.Protoc.* **2**: 727-738.

Keseler,I.M., Collado-Vides,J., Gama-Castro,S., Ingraham,J., Paley,S., Paulsen,I.T., Peralta-Gil,M., and Karp,P.D. (2005) EcoCyc: a comprehensive database resource for Escherichia coli *Nucleic Acids Res.* **33**: D334-D337.

Reed,J.L., Vo,T.D., Schilling,C.H., and Palsson,B.O. (2003) An expanded genome-scale model of Escherichia coli K-12 (iJR904 GSM/GPR) *Genome Biol* **4**: R54.

Zientz,E., Dandekar,T., and Gross,R. (2004) Metabolic interdependence of obligate intracellular bacteria and their insect hosts *Microbiol.Mol.Biol.Rev.* **68**: 745-770.
