# Supplementary material for: A fragile metabolic network adapted for cooperation in the symbiotic bacterium Buchnera aphidicola
Source: BMC Syst Biol. 2009 Feb 21;3:24. doi: 10.1186/1752-0509-3-24 (PMC2649895; doi:10.1186/1752-0509-3-24)
Supplement: Additional file 2 — Quantification of essential amino acid release from B. aphidicola APS. These data demonstrate the contribution of B. aphidicola APS-derived essential amino acids to protein growth of 2-to-7-day-old larval pea aphids (clone LL01) on chemically-defined diets. [file 1752-0509-3-24-S2.doc]

A fragile metabolic network adapted for cooperation in the symbiotic bacterium *Buchnera aphidicola*

Gavin H. Thomas^1*^, Jeremy Zucker^2*^, Sandy J Macdonald^1^, Anatoly Sorokin^3^, Igor Goryanin^3^ and Angela E. Douglas^1#^

**Additional File 2**

Additional File 2. Quantification of essential amino acid release from *B. aphidicola APS*

The contribution of *B. aphidicola APS-*derived essential amino acids to protein growth of 2-to-7-day-old larval pea aphids (clone LL01) on chemically-defined diets from which each essential amino acid was individually omitted was quantified(Douglas, Minto, and Wilkinson, 2001).

| Essential amino acid | Rate of increase in *B. aphidicola APS*-derived amino acids in aphid protein (pmol μg^-1^ protein day^-1^)^a^ | Rate of protein-amino acid release from *B. aphidicola APS* cells (fmol cell^-1^ day^-1^)^b^ | Rate of protein-amino acid increase in *B. aphidicola APS* cells (fmol cell^-1^ day^-1^)^c^ | %- amino acids released |
| --- | --- | --- | --- | --- |
| Histidine | 9.0 | 0.048 | 0.16 | 22 |
| Isoleucine | 80.9 | 0.428 | 0.622 | 41 |
| Leucine | 104.8 | 0.885 | 0.997 | 47 |
| Lysine | 167.2 | 0.550 | 0.729 | 43 |
| Methionine | 32.2 | 0.170 | 0.271 | 39 |
| Phenylalanine | 64.2 | 0.339 | 0.381 | 47 |
| Threonine | 99.2 | 0.525 | 0.523 | 50 |
| Tryptophan | 6.3 | 0.033 | 0.119 | 22 |
| Valine | 98.3 | 0.520 | 1.020 | 34 |

^a^ Derived from the difference between the protein growth rate of symbiotic aphids (containing *B. aphidicola APS*) and aposymbiotic aphids (experimentally deprived of *B. aphidicola APS* by antibiotic treatment) on diets from which each essential amino acid was individually omitted.

^b^ Calculated using the empirically-determined density of *B. aphidicola APS* population, 1.89 x 10^5^ cells μg^-1^ aphid protein

^c^ Calculated on the assumption that the *B. aphidicola APS* population increases proportionately with aphid growth, and using published values of *E. coli* amino acid content (Schaecter, 1992) as a best estimate of the amino acid content of *B. aphidicola APS* (corrected for difference in cell volume between *E. coli* and *B. aphidicola APS*).

Reference List for Additional Table 1

Douglas,A.E., Minto,L.B., and Wilkinson,T.L. (2001) Quantifying nutrient production by the microbial symbionts in an aphid *J Exp.Biol* **204**: 349-358.

Schaecter,M. (1992) In *Encyclopedia of Microbiology* . Lederberg,J. (ed). Academic Press, London, pp. 115-124.
