# Supplementary material for: A fragile metabolic network adapted for cooperation in the symbiotic bacterium Buchnera aphidicola
Source: BMC Syst Biol. 2009 Feb 21;3:24. doi: 10.1186/1752-0509-3-24 (PMC2649895; doi:10.1186/1752-0509-3-24)
Supplement: Additional file 4 — Analysis of the FBA simulations for non-essential genes. The file summarises the results from our analysis of the FBA simulations for non-essential genes from iGT196 by our own method and also by using the linearMOMA method. [file 1752-0509-3-24-S4.doc]

A fragile metabolic network adapted for cooperation in the symbiotic bacterium *Buchnera aphidicola*

Gavin H. Thomas^1*^, Jeremy Zucker^2*^, Sandy J Macdonald^1^, Anatoly Sorokin^3^, Igor Goryanin^3^ and Angela E. Douglas^1#^

Additional File 4

**Additional File 4**. Analysis of the FBA simulations for non-essential genes from iGT196 by our own method and also by using the linearMOMA method. The flux in the described 5.21 model is indicated with the flux when each particular gene is deleted. This is presented as a % decrease for both our method and the linearMOMA method.

| **Gene name** | **Reaction** | **Protein function** | **Flux in 5.21 model** | **Vgrowth when removed (FBA)** | **% of Vgrowth (our method)** | **% of Vgrowth (MOMA method)** |
| --- | --- | --- | --- | --- | --- | --- |
| *gpt* | GUAPRT | guanine phosphoribosyltransferase | 2.23 | 5.213355 | 100.00 | 100 |
| *hpt* | GUAPRT | guanine phosphoribosyltransferase | 2.23 | 5.213355 | 100.00 | 100 |
| *lpcA* | S7PI | phosphoheptose isomerase | 0 | 5.213355 | 100.00 | 100 |
| *rfaE* | GMHEPAT GMHEPK | D-glycero-D-manno-heptose 7-phosphate kinase  (GMHEPK) | 0 | 5.213355 | 100.00 | 100 |
| *dcd* | DCTPD | dCTP deaminase  (DCTPD) | 0.191867 | 5.211058 | 99.96 | 99.88 |
| *mtlA* | MNLptspp | PTS system mannitol-specific IIABC component | 6.977134 | 4.991764 | 95.75 | 95.27 |
| *mtlD* | M1PD | mannitol-1-phosphate 5-dehydrogenase | 6.977134 | 4.991764 | 95.75 | 95.27 |
| *pgi* | PGI | glucose-6-phosphate isomerase | 41.00292 | 4.983006 | 95.58 | 94.49 |
| *pykA* | PYK | pyruvate kinase | 60.66715 | 4.406621 | 84.53 | 82.99 |
| *ackA* | ACKr | acetate kinase | -55.9838 | 3.875808 | 74.34 | 72.14 |
| *pta* | PTAr | phosphate acetyltransferase | 55.9838 | 3.875808 | 74.34 | 72.14 |
| *crr* | GLCptspp | glucose-permease IIA component | 100 | 2.645691 | 50.75 | 48.85 |
| *pysG* | GLCptspp | PTS system glucose-specific IIBC component | 100 | 2.645691 | 50.75 | 48.85 |
| *rpe* | RPE | ribulose-phosphate 3-epimerase | 33.27528 | 1.044135 | 20.03 | 20.03 |
| *talA* | TALA | transaldolase A | 19.72188 | 0.936258 | 17.96 | 17.96 |
| *gnd* | GND | decarboxylating 6-phosphogluconate dehydrogenase | 58.99708 | 0.533129 | 10.23 | 10.23 |
| *pgl* | PGL | 6-phosphogluconolactonase | 58.99708 | 0.533129 | 10.23 | 10.23 |
| *zwf* | G6PDH2r | glucose-6-phosphate 1-dehydrogenase | 58.99708 | 0.533129 | 10.23 | 10.23 |
| *nuoA-N (13 genes)* | NADH6pp | NADH dehydrogenase I chains A-N | 34.238 | 0.055857 | 1.07 | 0 |
