# Supplementary material for: A fragile metabolic network adapted for cooperation in the symbiotic bacterium Buchnera aphidicola
Source: BMC Syst Biol. 2009 Feb 21;3:24. doi: 10.1186/1752-0509-3-24 (PMC2649895; doi:10.1186/1752-0509-3-24)

A fragile metabolic network adapted for cooperation in the symbiotic bacterium *Buchnera aphidicola*

Gavin H. Thomas^1*^, Jeremy Zucker^2*^, Sandy J Macdonald^1^, Anatoly Sorokin^3^, Igor Goryanin^3^ and Angela E. Douglas^1#^

Additional File 5

**Additional File 5 Legend**. Pairwise scatterplots showing correlation of fluxes in reactions of the histidine and purine biosynthetic pathways of *Buchnera aphidicola*. Line plots on the diagonal show the magnitude of flux through the respective reaction, whilst off-diagonal scatterplots show correlations between pairs of reactions. For each scatterplot, 100 points are shown from 20,000 flux distributions calculated by the software. Calculations assume optimal growth rate as determined from FBA. A positive correlation between a pair of reactions is inferred when the majority of points on a given scatterplot fall on a diagnonal line with positive gradient. Simulations were performed using COBRA toolbox (Becke*r et a*l., 2007) for Matlab (The Mathworks Inc.). Reaction abbreviations are: ADK1: adenylate kinase; ADSL1r: adenylosuccinate lyase; ADSS: adenylosuccinate synthase; AICART: phosphoribosylaminoimidazolecarboxamide formyltransferase; ATPPRT: ATP phosphoribosyltransferase; GMPR: GMP reductase; HISTD: histidinol dehydrogenase; HISTP: histidinol phosphatase; HSTPT: histidinol phosphatase; IG3PS: imidazole-glycerol-3-phosphate synthase; IGPDH: imidazole-glycerol-phosphate dehydratase; IMPC: IMP cyclohydrolase; NDPK8: nucleoside-diphosphate kinase (ATP:dADP); PRATPP: phosphoribosyl-ATP pyrophosphatase; PRMICI: 1-(5-phosphoribosyl)-5-(5-phosphoribosylamino)methylideneamino)imidazole-4-carboxamide isomerise; PRPPS: phosphoribosylpyrophosphate synthetase; RNDR1: ribonucleoside-diphosphate reductase (ADP).

Additional file 5


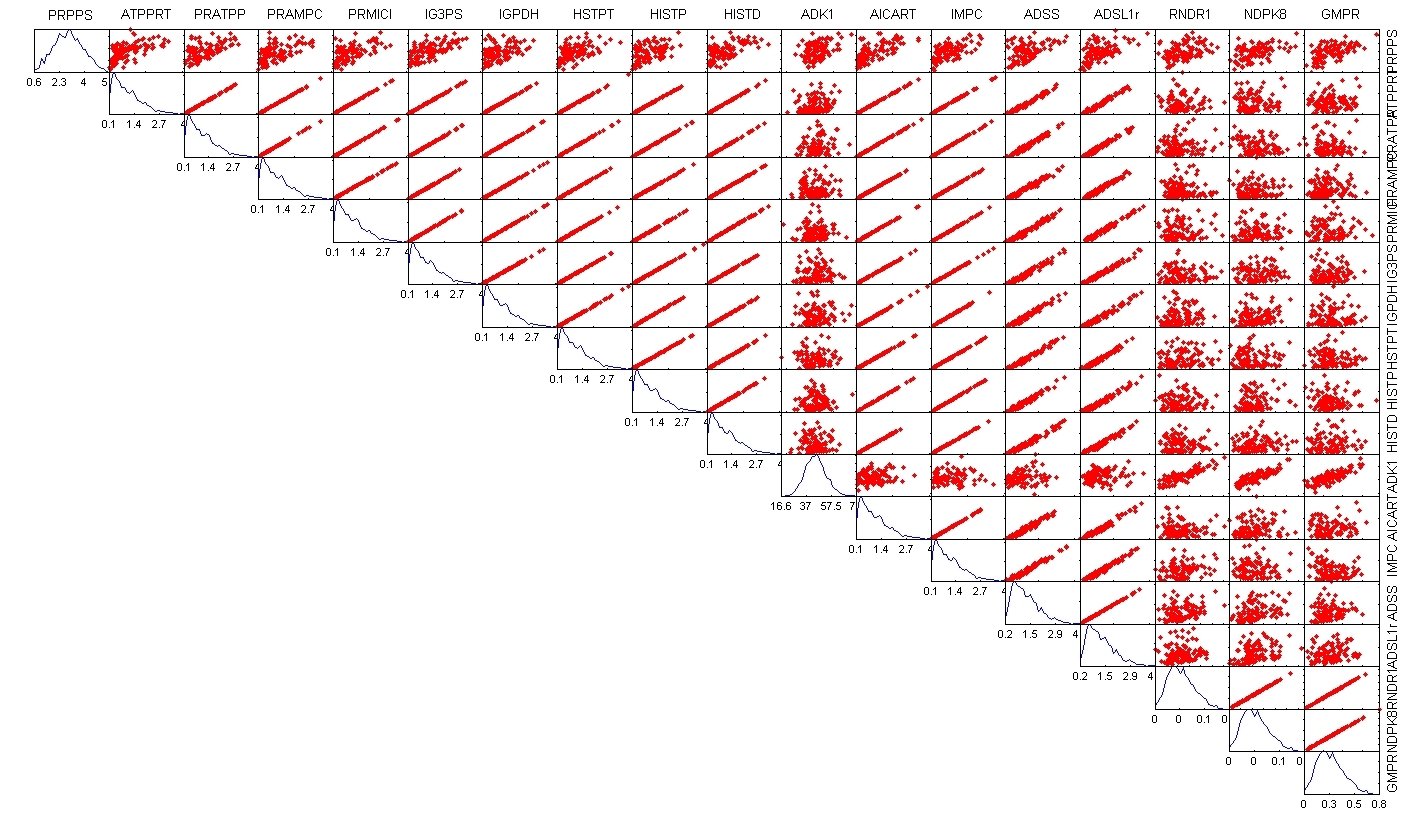

Supplement: Additional file 5 — Flux correlation of the histidine and purine biosynthesis pathways. Pairwise scatterplots showing correlation of fluxes in reactions of the histidine and purine biosynthetic pathways of Buchnera aphidicola. [file 1752-0509-3-24-S5.doc]
