# Supplementary material for: A fragile metabolic network adapted for cooperation in the symbiotic bacterium Buchnera aphidicola
Source: BMC Syst Biol. 2009 Feb 21;3:24. doi: 10.1186/1752-0509-3-24 (PMC2649895; doi:10.1186/1752-0509-3-24)
Supplement: Additional file 6 — List of APS genes and the frequency of their retention in evolved symbionts. This file lists the APS genes and the frequency of their retention in evolved symbionts from Pal et al,. (2006) and this study. [file 1752-0509-3-24-S6.doc]

A fragile metabolic network adapted for cooperation in the symbiotic bacterium *Buchnera aphidicola*

Gavin H. Thomas^1*^, Jeremy Zucker^2*^, Sandy J Macdonald^1^, Anatoly Sorokin^3^, Igor Goryanin^3^ and Angela E. Douglas^1#^

Additional File 6

List of APS genes and the frequency of their retention in evolved symbionts from Pal *et al*,*.* (2006)(Pa*l et a*l., 2006) and this study.

| **Blattner number** | **Gene name** | **Present in *Buchnera* sp. APS** | **Frequency of retention in Pal *et al*. Buchnera conditions** | **Frequency of retention in our simulations (of 500 runs)** |
| --- | --- | --- | --- | --- |
| b0002 | thrA | **+** | 0.498 | 0.474 |
| b0003 | thrB | **+** | 0.606 | 0.652 |
| b0004 | thrC | **+** | 0.606 | 0.652 |
| b0007 | yaaJ | **-** | 0 | 0.040 |
| b0008 | talB | **-** | 0.512 | 0.478 |
| b0019 | nhaA | **-** | 0 | 0.000 |
| b0025 | ribF | **+** | 1 | 1.000 |
| b0029 | lytB | **+** | 0 | 1.000 |
| b0031 | dapB | **+** | 1 | 1.000 |
| b0032 | carA | **+** | 0.012 | 0.000 |
| b0033 | carB | **+** | 0.012 | 0.000 |
| b0036 | caiD | **-** | 0 | 0.000 |
| b0038 | caiB | **-** | 0 | 0.000 |
| b0040 | caiT | **-** | 0 | 0.000 |
| b0048 | folA | **+** | 1 | 1.000 |
| b0049 | apaH | **+** | 0 | 0.000 |
| b0052 | pdxA | **-** | 0 | 0.000 |
| b0061 | araD | **-** | 0 | 0.004 |
| b0062 | araA | **-** | 0 | 0.000 |
| b0063 | araB | **-** | 0 | 0.000 |
| b0066 | sfuC | **-** | 0 | 0.000 |
| b0067 | sfuB | **-** | 0 | 0.000 |
| b0068 | sfuA | **-** | 0 | 0.000 |
| b0071 | leuD | **+** | 1 | 1.000 |
| b0072 | leuC | **+** | 1 | 1.000 |
| b0073 | leuB | **+** | 1 | 1.000 |
| b0074 | leuA | **+** | 1 | 1.000 |
| b0077 | ilvI | **+** | 0.414 | 0.383 |
| b0078 | ilvH | **+** | 0.414 | 0.383 |
| b0085 | murE | **+** | 1 | 1.000 |
| b0086 | murF | **+** | 1 | 1.000 |
| b0087 | mraY | **+** | 1 | 1.000 |
| b0088 | murD | **+** | 1 | 1.000 |
| b0090 | murG | **+** | 1 | 1.000 |
| b0091 | murC | **+** | 1 | 1.000 |
| b0092 | ddlB | **-** | 0.51 | 0.518 |
| b0096 | lpxC | **-** | 1 | 0.000 |
| b0099 | mutT | **+** | 0.48 | 0.506 |
| b0104 | guaC | **+** | 0 | 0.000 |
| b0109 | nadC | **-** | 1 | 1.000 |
| b0112 | aroP | **-** | 0 | 0.000 |
| b0114 | aceE | **+** | 1 | 1.000 |
| b0115 | aceF | **+** | 1 | 1.000 |
| b0116 | lpdA | **+** | 1 | 1.000 |
| b0118 | acnB | **-** | 0.484 | 0.486 |
| b0120 | speD | **+** | 1 | 1.000 |
| b0121 | speE | **+** | 1 | 1.000 |
| b0124 | gcd | **-** | 0 | 0.000 |
| b0125 | hpt | **+** | 0.054 | 0.043 |
| b0126 | yadF | **-** | 0.536 | 0.474 |
| b0131 | panD | **-** | 1 | 1.000 |
| b0133 | panC | **+** | 1 | 1.000 |
| b0134 | panB | **+** | 1 | 1.000 |
| b0142 | folK | **-** | 1 | 1.000 |
| b0154 | hemL | **-** | 0 | 1.000 |
| b0158 | yadT | **-** | 0 | 0.000 |
| b0159 | mtn | **+** | 1 | 1.000 |
| b0160 | dgt | **-** | 0 | 0.000 |
| b0166 | dapD | **+** | 1 | 1.000 |
| b0171 | pyrH | **+** | 0.618 | 0.494 |
| b0173 | dxr | **+** | 0 | 1.000 |
| b0174 | uppS | **+** | 0 | 1.000 |
| b0175 | cdsA | **-** | 1 | 1.000 |
| b0179 | lpxD | **-** | 1 | 0.004 |
| b0180 | fabZ | **-** | 1 | 1.000 |
| b0181 | lpxA | **-** | 1 | 0.004 |
| b0182 | lpxB | **-** | 1 | 0.004 |
| b0185 | accA | **-** | 1 | 1.000 |
| b0186 | ldcC | **-** | 0 | 0.000 |
| b0197 | yaeC | **-** | 0 | 0.000 |
| b0198 | yaeE | **-** | 0 | 0.000 |
| b0199 | abc | **-** | 0 | 0.000 |
| b0200 | yaeD | **-** | 1 | 0.000 |
| b0207 | yafB | **-** | 0 | 0.000 |
| b0212 | gloB | **+** | 0 | 0.000 |
| b0221 | fadF | **-** | 0 | 0.000 |
| b0222 | gmhA | **+** | 1 | 0.000 |
| b0238 | gpt | **+** | 0.046 | 0.047 |
| b0242 | proB | **-** | 0.388 | 0.000 |
| b0243 | proA | **-** | 0.388 | 0.000 |
| b0273 | argF | **+** | 0.502 | 0.514 |
| b0312 | betB | **-** | 0 | 0.000 |
| b0314 | betT | **-** | 0 | 0.000 |
| b0323 | yahI | **-** | 0.302 | 0.348 |
| b0331 | prpB | **-** | 0.002 | 0.000 |
| b0333 | prpC | **-** | 0.002 | 0.000 |
| b0334 | prpD | **-** | 0.002 | 0.000 |
| b0335 | prpE | **-** | 0.014 | 0.455 |
| b0336 | codB | **-** | 0 | 0.000 |
| b0337 | codA | **-** | 0.122 | 0.051 |
| b0339 | cynT | **-** | 0.464 | 0.526 |
| b0340 | cynS | **-** | 0 | 0.000 |
| b0341 | cynX | **-** | 0 | 0.000 |
| b0343 | lacY | **-** | 0 | 0.000 |
| b0344 | lacZ | **-** | 0 | 0.000 |
| b0347 | mhpA | **-** | 0 | 0.000 |
| b0348 | mhpB | **-** | 0 | 0.000 |
| b0349 | mhpC | **-** | 0 | 0.000 |
| b0350 | mhpD | **-** | 0 | 0.000 |
| b0351 | mhpF | **-** | 0.044 | 0.012 |
| b0352 | mhpE | **-** | 0 | 0.000 |
| b0353 | mhpT | **-** | 0 | 0.004 |
| b0356 | adhC | **-** | 0.008 | 0.000 |
| b0365 | tauA | **-** | 0 | 0.000 |
| b0366 | tauB | **-** | 0 | 0.000 |
| b0367 | tauC | **-** | 0 | 0.000 |
| b0368 | tauD | **-** | 0 | 0.000 |
| b0369 | hemB | **-** | 0 | 1.000 |
| b0381 | ddlA | **-** | 0.49 | 0.482 |
| b0386 | proC | **-** | 1 | 0.000 |
| b0388 | aroL | **-** | 0.52 | 0.526 |
| b0401 | brnQ | **-** | 0 | 0.000 |
| b0403 | malZ | **-** | 0 | 0.000 |
| b0414 | ribD | **+** | 1 | 1.000 |
| b0415 | ribH | **+** | 1 | 1.000 |
| b0417 | thiL | **+** | 0 | 1.000 |
| b0418 | pgpA | **-** | 0.46 | 0.474 |
| b0420 | dxs | **+** | 0 | 1.000 |
| b0421 | ispA | **+** | 0 | 1.000 |
| b0423 | thiI | **-** | 0 | 0.170 |
| b0425 | panE | **-** | 0 | 0.000 |
| b0428 | cyoE | **+** | 0 | 1.000 |
| b0429 | cyoD | **+** | 1 | 1.000 |
| b0430 | cyoC | **+** | 1 | 1.000 |
| b0431 | cyoB | **+** | 1 | 1.000 |
| b0432 | cyoA | **+** | 1 | 1.000 |
| b0451 | amtB | **-** | 1 | 1.000 |
| b0469 | apt | **-** | 0.126 | 0.206 |
| b0474 | adk | **+** | 1 | 1.000 |
| b0475 | hemH | **-** | 0 | 1.000 |
| b0477 | gsk | **-** | 0 | 0.004 |
| b0480 | ushA | **-** | 1 | 0.510 |
| b0485 | ybaS | **-** | 0 | 0.000 |
| b0505 | allA | **-** | 0 | 0.004 |
| b0507 | gcl | **-** | 0.014 | 0.040 |
| b0508 | hyi | **-** | 0.004 | 0.000 |
| b0509 | glxR | **-** | 0.008 | 0.024 |
| b0511 | allP | **-** | 0 | 0.000 |
| b0512 | allB | **-** | 0 | 0.000 |
| b0514 | glxK | **-** | 0.012 | 0.024 |
| b0516 | allC | **-** | 0 | 0.000 |
| b0521 | arcC | **-** | 0.354 | 0.344 |
| b0522 | purK | **-** | 1 | 1.000 |
| b0523 | purE | **-** | 1 | 1.000 |
| b0529 | folD | **+** | 1 | 1.000 |
| b0576 | pheP | **-** | 0 | 0.000 |
| b0583 | entD | **-** | 0 | 0.000 |
| b0586 | entF | **-** | 0 | 0.004 |
| b0593 | entC | **-** | 0 | 0.000 |
| b0594 | entE | **-** | 0 | 0.000 |
| b0595 | entB | **-** | 0 | 0.000 |
| b0596 | entA | **-** | 0 | 0.000 |
| b0612 | citT | **-** | 0 | 0.000 |
| b0615 | citF | **-** | 0 | 0.000 |
| b0616 | citE | **-** | 0 | 0.000 |
| b0617 | citD | **-** | 0 | 0.000 |
| b0621 | dcuC | **-** | 0 | 0.020 |
| b0638 | phpB | **-** | 0 | 0.000 |
| b0639 | nadD | **+** | 1 | 1.000 |
| b0652 | gltL | **-** | 0 | 0.000 |
| b0653 | gltK | **-** | 0 | 0.000 |
| b0654 | gltJ | **-** | 0 | 0.000 |
| b0655 | gltI | **-** | 0 | 0.000 |
| b0662 | ubiF | **-** | 0 | 0.000 |
| b0674 | asnB | **-** | 0.306 | 0.000 |
| b0677 | nagA | **-** | 0 | 0.000 |
| b0678 | nagB | **+** | 0 | 0.000 |
| b0679 | nagE | **-** | 0 | 0.000 |
| b0688 | pgm | **-** | 0.476 | 0.000 |
| b0692 | potE | **-** | 0 | 0.000 |
| b0693 | speF | **-** | 0.45 | 0.427 |
| b0696 | kdpC | **-** | 0 | 0.000 |
| b0697 | kdpB | **-** | 0 | 0.000 |
| b0698 | kdpA | **-** | 0 | 0.000 |
| b0720 | gltA | **-** | 1 | 1.000 |
| b0721 | sdhC | **-** | 1 | 0.028 |
| b0722 | sdhD | **-** | 1 | 0.028 |
| b0723 | sdhA | **-** | 1 | 0.028 |
| b0724 | sdhB | **-** | 1 | 0.028 |
| b0726 | sucA | **+** | 1 | 0.028 |
| b0727 | sucB | **+** | 1 | 0.028 |
| b0728 | sucC | **-** | 0.692 | 0.518 |
| b0729 | sucD | **-** | 0.692 | 0.518 |
| b0733 | cydA | **-** | 0 | 0.000 |
| b0734 | cydB | **-** | 0 | 0.000 |
| b0750 | nadA | **-** | 1 | 1.000 |
| b0751 | pnuC | **-** | 0 | 0.000 |
| b0754 | aroG | **-** | 0.326 | 0.332 |
| b0755 | gpmA | **+** | 0.32 | 0.320 |
| b0757 | galK | **-** | 0 | 0.004 |
| b0758 | galT | **-** | 0 | 0.000 |
| b0759 | galE | **-** | 0 | 0.000 |
| b0774 | bioA | **+** | 0 | 1.000 |
| b0775 | bioB | **+** | 0 | 1.000 |
| b0776 | bioF | **-** | 0 | 1.000 |
| b0778 | bioD | **+** | 0 | 1.000 |
| b0809 | glnQ | **-** | 0 | 0.000 |
| b0810 | glnP | **-** | 0 | 0.000 |
| b0811 | glnH | **-** | 0 | 0.000 |
| b0825 | fsa | **-** | 0.138 | 0.178 |
| b0828 | ybiK | **-** | 0 | 0.000 |
| b0854 | potF | **-** | 0 | 0.000 |
| b0855 | potG | **-** | 0 | 0.000 |
| b0856 | potH | **-** | 0 | 0.000 |
| b0857 | potI | **-** | 0 | 0.000 |
| b0860 | artJ | **-** | 0 | 0.000 |
| b0861 | artM | **-** | 0 | 0.000 |
| b0862 | artQ | **-** | 0 | 0.000 |
| b0864 | artP | **-** | 0 | 0.000 |
| b0870 | ltaA | **-** | 0 | 0.000 |
| b0871 | poxB | **-** | 0 | 0.000 |
| b0888 | trxB | **+** | 1 | 1.000 |
| b0894 | dmsA | **-** | 0 | 0.000 |
| b0895 | dmsB | **-** | 0 | 0.000 |
| b0896 | dmsC | **-** | 0 | 0.000 |
| b0902 | pflA | **-** | 0.064 | 0.194 |
| b0903 | pflB | **-** | 0.064 | 0.194 |
| b0904 | focA | **-** | 0.08 | 0.447 |
| b0907 | serC | **+** | 1 | 1.000 |
| b0908 | aroA | **+** | 1 | 1.000 |
| b0910 | cmk | **-** | 0.792 | 1.000 |
| b0915 | lpxK | **-** | 1 | 0.000 |
| b0918 | kdsB | **-** | 1 | 0.000 |
| b0928 | aspC | **-** | 1 | 1.000 |
| b0931 | pncB | **+** | 0 | 0.000 |
| b0945 | pyrD | **+** | 1 | 1.000 |
| b0954 | fabA | **-** | 1 | 1.000 |
| b0963 | mgsA | **-** | 0 | 0.000 |
| b0972 | hyaA | **-** | 0 | 0.000 |
| b0973 | hyaB | **-** | 0 | 0.000 |
| b0974 | hyaC | **-** | 0 | 0.000 |
| b0996 | torC | **-** | 0 | 0.000 |
| b0997 | torA | **-** | 0 | 0.000 |
| b1002 | agp | **-** | 0 | 0.000 |
| b1006 | ycdG | **-** | 0 | 0.000 |
| b1014 | putA | **-** | 0 | 0.004 |
| b1015 | putP | **-** | 0 | 0.000 |
| b1033 | ycdW | **-** | 0.002 | 0.000 |
| b1054 | lpxL | **-** | 1 | 0.000 |
| b1062 | pyrC | **+** | 1 | 1.000 |
| b1091 | fabH | **-** | 0.558 | 1.000 |
| b1092 | fabD | **-** | 1 | 1.000 |
| b1093 | fabG | **+** | 1 | 1.000 |
| b1095 | fabF | **-** | 1 | 1.000 |
| b1096 | pabC | **-** | 1 | 1.000 |
| b1098 | tmk | **+** | 1 | 1.000 |
| b1101 | ptsG | **+** | 0.374 | 0.348 |
| b1109 | ndh | **-** | 0 | 0.000 |
| b1123 | potD | **-** | 0 | 0.000 |
| b1124 | potC | **-** | 0 | 0.000 |
| b1125 | potB | **-** | 0 | 0.000 |
| b1126 | potA | **-** | 0 | 0.000 |
| b1131 | purB | **+** | 1 | 1.000 |
| b1136 | icdA | **-** | 1 | 1.000 |
| b1186 | nhaB | **-** | 0.002 | 0.000 |
| b1189 | dadA | **-** | 0 | 0.000 |
| b1190 | dadX | **-** | 0.528 | 0.557 |
| b1197 | treA | **-** | 0 | 0.000 |
| b1198 | dhaH | **-** | 0.106 | 0.107 |
| b1199 | dhaK2 | **-** | 0.106 | 0.107 |
| b1200 | dhaK1 | **-** | 0.106 | 0.107 |
| b1207 | prsA | **+** | 1 | 1.000 |
| b1208 | ispE | **+** | 0 | 1.000 |
| b1210 | hemA | **-** | 0 | 1.000 |
| b1215 | kdsA | **-** | 1 | 0.000 |
| b1216 | chaA | **-** | 0.004 | 0.000 |
| b1223 | narK | **-** | 0.002 | 0.000 |
| b1224 | narG | **-** | 0 | 0.000 |
| b1225 | narH | **-** | 0 | 0.000 |
| b1226 | narJ | **-** | 0 | 0.000 |
| b1227 | narI | **-** | 0 | 0.000 |
| b1232 | purU | **-** | 0.494 | 0.435 |
| b1236 | galU | **-** | 0.526 | 0.000 |
| b1238 | tdk | **-** | 0.13 | 0.166 |
| b1241 | adhE | **-** | 0 | 0.000 |
| b1249 | cls | **+** | 1 | 1.000 |
| b1260 | trpA | **+** | 1 | 1.000 |
| b1261 | trpB | **+** | 1 | 1.000 |
| b1262 | trpC | **+** | 1 | 1.000 |
| b1263 | trpD | **+** | 1 | 1.000 |
| b1264 | trpE | **+** | 1 | 1.000 |
| b1270 | btuR | **-** | 0 | 0.004 |
| b1276 | acnA | **-** | 0.516 | 0.514 |
| b1277 | ribA | **+** | 1 | 1.000 |
| b1278 | pgpB | **-** | 0.54 | 0.522 |
| b1281 | pyrF | **+** | 1 | 1.000 |
| b1288 | fabI | **+** | 1 | 1.000 |
| b1297 | ycjK | **-** | 0.51 | 0.522 |
| b1300 | aldH | **-** | 0.002 | 0.000 |
| b1302 | goaG | **-** | 0.058 | 0.000 |
| b1363 | trkG | **-** | 0 | 0.000 |
| b1380 | ldhA | **-** | 0 | 0.000 |
| b1385 | feaB | **-** | 0 | 0.000 |
| b1386 | tynA | **-** | 0 | 0.000 |
| b1398 | paaK | **-** | 0 | 0.000 |
| b1415 | aldA | **-** | 1 | 1.000 |
| b1416 | gapC_2 | **?** | 0.298 | 0.316 |
| b1417 | gapC_1 | **?** | 0.298 | 0.316 |
| b1440 | ydcS | **-** | 0 | 0.000 |
| b1441 | ydcT | **-** | 0 | 0.000 |
| b1442 | ydcU | **-** | 0 | 0.000 |
| b1443 | ydcV | **-** | 0 | 0.000 |
| b1469 | narU | **-** | 0 | 0.000 |
| b1474 | fdnG | **-** | 0.082 | 0.004 |
| b1475 | fdnH | **-** | 0.082 | 0.004 |
| b1476 | fdnI | **-** | 0.082 | 0.004 |
| b1479 | sfcA | **-** | 0.044 | 0.004 |
| b1492 | xasA | **-** | 0.006 | 0.000 |
| b1493 | gadB | **-** | 0.056 | 0.000 |
| b1519 | tam | **-** | 0 | 0.000 |
| b1521 | uxaB | **-** | 0 | 0.000 |
| b1524 | yneH | **-** | 0 | 0.000 |
| b1584 | speG | **-** | 0 | 0.008 |
| b1602 | pntB | **-** | 0.03 | 0.245 |
| b1603 | pntA | **-** | 0.03 | 0.245 |
| b1605 | arcD | **-** | 0 | 0.000 |
| b1611 | fumC | **-** | 0.338 | 0.332 |
| b1612 | fumA | **-** | 0.342 | 0.324 |
| b1613 | manA | **-** | 0 | 0.000 |
| b1621 | malX | **-** | 0.328 | 0.308 |
| b1622 | malY | **-** | 0.49 | 0.490 |
| b1623 | add | **-** | 0 | 0.028 |
| b1636 | pdxY | **-** | 0 | 0.000 |
| b1638 | pdxH | **-** | 0 | 0.000 |
| b1646 | sodC | **-** | 0 | 0.004 |
| b1651 | gloA | **-** | 0 | 0.000 |
| b1656 | sodB | **-** | 0 | 0.000 |
| b1662 | ribE | **+** | 1 | 1.000 |
| b1676 | pykF | **-** | 0.362 | 0.439 |
| b1692 | ydiB | **-** | 0.466 | 0.518 |
| b1693 | aroD | **-** | 1 | 1.000 |
| b1702 | pps | **-** | 0 | 0.004 |
| b1704 | aroH | **+** | 0.34 | 0.375 |
| b1709 | btuD | **-** | 0 | 0.000 |
| b1711 | btuC | **-** | 0 | 0.000 |
| b1723 | pfkB | **-** | 0.44 | 0.553 |
| b1732 | katE | **-** | 0 | 0.000 |
| b1740 | nadE | **+** | 1 | 1.000 |
| b1744 | astE | **-** | 0 | 0.000 |
| b1745 | astB | **-** | 0 | 0.000 |
| b1746 | astD | **-** | 0 | 0.000 |
| b1747 | astA | **-** | 0 | 0.004 |
| b1748 | astC | **-** | 0 | 0.004 |
| b1761 | gdhA | **-** | 1 | 1.000 |
| b1764 | selD | **-** | 0 | 0.000 |
| b1767 | ansA | **-** | 0 | 0.000 |
| b1768 | pncA | **-** | 0 | 0.000 |
| b1773 | b1773 | **-** | 0.348 | 0.352 |
| b1779 | gapA | **+** | 0.702 | 0.684 |
| b1801 | yeaV | **-** | 0.012 | 0.051 |
| b1805 | fadD | **-** | 0 | 0.000 |
| b1812 | pabB | **-** | 1 | 1.000 |
| b1814 | sdaA | **-** | 0.258 | 0.225 |
| b1817 | manX | **-** | 0.298 | 0.344 |
| b1818 | manY | **-** | 0.298 | 0.344 |
| b1819 | manZ | **-** | 0.298 | 0.344 |
| b1849 | purT | **-** | 0.568 | 0.613 |
| b1850 | eda | **-** | 0 | 0.016 |
| b1851 | edd | **-** | 0 | 0.016 |
| b1852 | zwf | **+** | 1 | 1.000 |
| b1854 | pykA | **+** | 0.42 | 0.478 |
| b1855 | msbB | **-** | 1 | 0.000 |
| b1865 | ntpA | **-** | 0.52 | 0.494 |
| b1872 | torZ | **-** | 0 | 0.000 |
| b1873 | torY | **-** | 0 | 0.000 |
| b1896 | otsA | **-** | 0 | 0.000 |
| b1897 | otsB | **-** | 0 | 0.000 |
| b1898 | araH_2 | **-** | 0 | 0.000 |
| b1899 | araH_1 | **-** | 0 | 0.000 |
| b1900 | araG | **-** | 0 | 0.000 |
| b1901 | araF | **-** | 0 | 0.000 |
| b1907 | tyrP | **-** | 0 | 0.004 |
| b1912 | pgsA | **-** | 1 | 1.000 |
| b1982 | amn | **-** | 0.002 | 0.000 |
| b1991 | cobT | **-** | 0 | 0.000 |
| b1992 | cobS | **-** | 0 | 0.000 |
| b1993 | cobU | **-** | 0 | 0.000 |
| b2019 | hisG | **+** | 1 | 1.000 |
| b2020 | hisD | **+** | 1 | 1.000 |
| b2021 | hisC | **+** | 1 | 1.000 |
| b2022 | hisB | **+** | 1 | 1.000 |
| b2023 | hisH | **+** | 1 | 1.000 |
| b2024 | hisA | **+** | 1 | 1.000 |
| b2025 | hisF | **+** | 1 | 1.000 |
| b2026 | hisI | **+** | 1 | 1.000 |
| b2028 | ugd | **-** | 0 | 0.000 |
| b2029 | gnd | **+** | 1 | 1.000 |
| b2036 | glf | **-** | 0 | 0.000 |
| b2038 | rfbC | **-** | 0 | 0.000 |
| b2039 | rfbA | **-** | 0 | 0.000 |
| b2040 | rfbD | **-** | 0 | 0.000 |
| b2041 | rfbB | **-** | 0 | 0.000 |
| b2042 | galF | **-** | 0.474 | 0.000 |
| b2045 | wcaK | **-** | 0 | 0.000 |
| b2048 | cpsG | **-** | 0 | 0.000 |
| b2049 | manC | **-** | 0 | 0.000 |
| b2052 | fcl | **-** | 0 | 0.000 |
| b2053 | gmd | **-** | 0 | 0.000 |
| b2065 | dcd | **+** | 0 | 0.000 |
| b2066 | udk | **-** | 0.142 | 0.036 |
| b2091 | gatD | **-** | 0 | 0.004 |
| b2092 | gatC | **-** | 0 | 0.000 |
| b2093 | gatB | **-** | 0 | 0.000 |
| b2094 | gatA | **-** | 0 | 0.000 |
| b2095 | gatZ | **-** | 0 | 0.000 |
| b2096 | gatY | **-** | 0 | 0.000 |
| b2097 | fbaB | **-** | 0.322 | 0.328 |
| b2103 | thiD | **-** | 0 | 1.000 |
| b2104 | thiM | **-** | 0 | 0.830 |
| b2128 | yehW | **-** | 0 | 0.000 |
| b2129 | yehX | **-** | 0 | 0.000 |
| b2130 | yehY | **-** | 0 | 0.000 |
| b2131 | yehZ | **-** | 0 | 0.000 |
| b2132 | bglX | **-** | 0 | 0.000 |
| b2133 | dld | **-** | 0 | 0.000 |
| b2143 | cdd | **-** | 0.172 | 0.067 |
| b2148 | mglC | **-** | 0 | 0.000 |
| b2149 | mglA | **-** | 0 | 0.000 |
| b2150 | mglB | **-** | 0 | 0.000 |
| b2153 | folE | **-** | 1 | 1.000 |
| b2156 | lysP | **-** | 0 | 0.000 |
| b2167 | fruA | **-** | 0 | 0.000 |
| b2168 | fruK | **-** | 0 | 0.000 |
| b2169 | fruB | **-** | 0 | 0.000 |
| b2210 | mqo | **-** | 0 | 0.083 |
| b2221 | atoD | **-** | 0 | 0.000 |
| b2222 | atoA | **-** | 0 | 0.000 |
| b2223 | atoE | **-** | 0 | 0.000 |
| b2224 | atoB | **-** | 0 | 0.004 |
| b2232 | ubiG | **-** | 0 | 0.000 |
| b2234 | nrdA | **+** | 0.312 | 0.348 |
| b2235 | nrdB | **+** | 0.312 | 0.348 |
| b2239 | glpQ | **-** | 0.006 | 0.000 |
| b2240 | glpT | **-** | 0 | 0.000 |
| b2241 | glpA | **-** | 0 | 0.000 |
| b2242 | glpB | **-** | 0 | 0.000 |
| b2243 | glpC | **-** | 0 | 0.000 |
| b2260 | menE | **-** | 0 | 0.000 |
| b2261 | menC | **-** | 0 | 0.000 |
| b2262 | menB | **-** | 0 | 0.000 |
| b2264 | menD | **-** | 0 | 0.000 |
| b2265 | menF | **-** | 0 | 0.000 |
| b2276 | nuoN | **+** | 1 | 1.000 |
| b2277 | nuoM | **+** | 1 | 0.996 |
| b2278 | nuoL | **+** | 1 | 1.000 |
| b2279 | nuoK | **+** | 1 | 1.000 |
| b2280 | nuoJ | **+** | 1 | 1.000 |
| b2281 | nuoI | **+** | 1 | 1.000 |
| b2282 | nuoH | **+** | 1 | 1.000 |
| b2283 | nuoG | **+** | 1 | 1.000 |
| b2284 | nuoF | **+** | 1 | 1.000 |
| b2285 | nuoE | **+** | 1 | 1.000 |
| b2286 | nuoC | **+** | 1 | 1.000 |
| b2287 | nuoB | **+** | 1 | 1.000 |
| b2288 | nuoA | **+** | 1 | 1.000 |
| b2296 | ackA | **+** | 0.16 | 0.198 |
| b2297 | pta | **+** | 0.466 | 0.542 |
| b2306 | hisP | **-** | 0 | 0.000 |
| b2307 | hisM | **-** | 0 | 0.000 |
| b2308 | hisQ | **-** | 0 | 0.000 |
| b2309 | hisJ | **-** | 0 | 0.000 |
| b2310 | argT | **-** | 0 | 0.000 |
| b2311 | ubiX | **-** | 0 | 0.000 |
| b2312 | purF | **-** | 1 | 1.000 |
| b2315 | folC | **+** | 1 | 1.000 |
| b2316 | accD | **-** | 1 | 1.000 |
| b2320 | pdxB | **-** | 0 | 0.004 |
| b2323 | fabB | **+** | 1 | 1.000 |
| b2329 | aroC | **+** | 1 | 1.000 |
| b2344 | fadL | **-** | 0 | 0.000 |
| b2366 | dsdA | **-** | 0 | 0.000 |
| b2378 | lpxP | **-** | 0 | 0.000 |
| b2388 | glk | **-** | 0.026 | 0.000 |
| b2393 | nupC | **-** | 0 | 0.000 |
| b2400 | gltX | **+** | 0 | 1.000 |
| b2406 | xapB | **-** | 0 | 0.000 |
| b2407 | xapA | **-** | 0.38 | 0.324 |
| b2411 | lig | **+** | 0 | 0.000 |
| b2413 | cysZ | **-** | 0.348 | 0.332 |
| b2414 | cysK | **+** | 0.514 | 0.490 |
| b2415 | ptsH | **+** | 1 | 1.000 |
| b2416 | ptsI | **+** | 1 | 1.000 |
| b2417 | crr | **+** | 0.702 | 0.656 |
| b2418 | pdxK | **-** | 0 | 0.000 |
| b2421 | cysM | **-** | 0.486 | 0.510 |
| b2422 | cysA | **-** | 1 | 1.000 |
| b2423 | cysW | **-** | 1 | 1.000 |
| b2424 | cysU | **-** | 1 | 1.000 |
| b2425 | cysP | **-** | 0.652 | 0.668 |
| b2429 | yfeV | **-** | 0 | 0.000 |
| b2436 | hemF | **-** | 0 | 1.000 |
| b2440 | eutC | **-** | 0.006 | 0.000 |
| b2441 | eutB | **-** | 0.006 | 0.000 |
| b2458 | eutD | **-** | 0.422 | 0.458 |
| b2463 | maeB | **-** | 0.094 | 0.249 |
| b2464 | talA | **+** | 0.482 | 0.522 |
| b2465 | tktB | **-** | 0.53 | 0.549 |
| b2472 | dapE | **+** | 1 | 1.000 |
| b2476 | purC | **-** | 1 | 1.000 |
| b2478 | dapA | **+** | 1 | 1.000 |
| b2492 | focB | **-** | 0.072 | 0.399 |
| b2497 | uraA | **-** | 0 | 0.000 |
| b2498 | upp | **-** | 0.118 | 0.004 |
| b2499 | purM | **-** | 1 | 1.000 |
| b2500 | purN | **-** | 0.736 | 0.652 |
| b2507 | guaA | **-** | 1 | 1.000 |
| b2508 | guaB | **-** | 1 | 1.000 |
| b2515 | gcpE | **+** | 0 | 1.000 |
| b2518 | ndk | **-** | 1 | 1.000 |
| b2530 | iscS | **+** | 0 | 0.170 |
| b2533 | suhB | **+** | 0 | 0.000 |
| b2536 | hcaT | **-** | 0 | 0.000 |
| b2538 | hcaE | **-** | 0 | 0.000 |
| b2539 | hcaF | **-** | 0 | 0.000 |
| b2540 | hcaC | **-** | 0 | 0.000 |
| b2541 | hcaB | **-** | 0 | 0.000 |
| b2542 | hcaD | **-** | 0 | 0.000 |
| b2551 | glyA | **+** | 1 | 1.000 |
| b2557 | purL | **-** | 1 | 1.000 |
| b2563 | acpS | **+** | 0 | 1.000 |
| b2564 | pdxJ | **-** | 0 | 0.000 |
| b2574 | nadB | **-** | 1 | 1.000 |
| b2585 | pssA | **-** | 1 | 0.000 |
| b2587 | kgtP | **-** | 0 | 0.000 |
| b2599 | pheA | **+** | 1 | 1.000 |
| b2600 | tyrA | **-** | 1 | 0.170 |
| b2601 | aroF | **-** | 0.334 | 0.292 |
| b2615 | yfjB | **+** | 1 | 1.000 |
| b2661 | gabD | **-** | 0.064 | 0.000 |
| b2662 | gabT | **-** | 0.052 | 0.000 |
| b2663 | gabP | **-** | 0.006 | 0.000 |
| b2675 | nrdE | **-** | 0 | 0.000 |
| b2676 | nrdF | **-** | 0 | 0.000 |
| b2677 | proV | **-** | 0 | 0.000 |
| b2678 | proW | **-** | 0 | 0.000 |
| b2679 | proX | **-** | 0 | 0.000 |
| b2687 | luxS | **-** | 0 | 0.000 |
| b2688 | gshA | **+** | 0 | 1.000 |
| b2690 | yqaB | **-** | 0.524 | 0.000 |
| b2702 | srlA | **-** | 0 | 0.000 |
| b2703 | srlE | **-** | 0 | 0.000 |
| b2704 | srlB | **-** | 0 | 0.000 |
| b2705 | srlD | **-** | 0 | 0.000 |
| b2719 | hycG | **-** | 0 | 0.000 |
| b2720 | hycF | **-** | 0 | 0.000 |
| b2721 | hycE | **-** | 0 | 0.000 |
| b2722 | hycD | **-** | 0 | 0.000 |
| b2723 | hycC | **-** | 0 | 0.000 |
| b2724 | hycB | **-** | 0 | 0.000 |
| b2738 | ygbL | **-** | 0 | 0.004 |
| b2746 | ispF | **+** | 0 | 1.000 |
| b2747 | ispD | **+** | 0 | 1.000 |
| b2750 | cysC | **+** | 1 | 1.000 |
| b2751 | cysN | **+** | 1 | 1.000 |
| b2752 | cysD | **+** | 1 | 1.000 |
| b2762 | cysH | **+** | 1 | 1.000 |
| b2763 | cysI | **+** | 1 | 1.000 |
| b2764 | cysJ | **+** | 1 | 1.000 |
| b2779 | eno | **+** | 1 | 1.000 |
| b2780 | pyrG | **+** | 1 | 1.000 |
| b2781 | mazG | **-** | 0 | 0.000 |
| b2787 | gudD | **-** | 0 | 0.000 |
| b2788 | ygcY | **-** | 0 | 0.000 |
| b2789 | gudP | **-** | 0 | 0.004 |
| b2796 | sdaC | **-** | 0 | 0.000 |
| b2797 | sdaB | **-** | 0.262 | 0.241 |
| b2799 | fucO | **-** | 0 | 0.000 |
| b2800 | fucA | **-** | 0 | 0.000 |
| b2801 | fucP | **-** | 0 | 0.004 |
| b2802 | fucI | **-** | 0 | 0.000 |
| b2803 | fucK | **-** | 0 | 0.000 |
| b2818 | argA | **+** | 1 | 1.000 |
| b2827 | thyA | **+** | 1 | 1.000 |
| b2836 | aas | **-** | 0.006 | 0.000 |
| b2838 | lysA | **+** | 1 | 1.000 |
| b2841 | araE | **-** | 0 | 0.004 |
| b2874 | yqeA | **-** | 0.332 | 0.308 |
| b2883 | ygfP | **-** | 0 | 0.000 |
| b2889 | idi | **-** | 0 | 0.000 |
| b2901 | bglA | **-** | 0 | 0.000 |
| b2903 | gcvP | **-** | 0.11 | 0.043 |
| b2904 | gcvH | **-** | 0.11 | 0.043 |
| b2905 | gcvT | **-** | 0.11 | 0.043 |
| b2907 | ubiH | **-** | 0 | 0.000 |
| b2913 | serA | **-** | 1 | 1.000 |
| b2914 | rpiA | **+** | 0.462 | 0.526 |
| b2917 | sbm | **-** | 0.002 | 0.000 |
| b2919 | ygfG | **-** | 0.002 | 0.000 |
| b2920 | ygfH | **-** | 0.014 | 0.455 |
| b2925 | fbaA | **+** | 0.304 | 0.320 |
| b2926 | pgk | **+** | 1 | 1.000 |
| b2927 | epd | **-** | 0 | 0.000 |
| b2935 | tktA | **+** | 0.47 | 0.451 |
| b2937 | speB | **-** | 0.098 | 0.150 |
| b2938 | speA | **-** | 0.064 | 0.079 |
| b2942 | metK | **+** | 1 | 1.000 |
| b2943 | galP | **-** | 0.026 | 0.000 |
| b2947 | gshB | **+** | 0 | 1.000 |
| b2957 | ansB | **-** | 0 | 0.000 |
| b2964 | nupG | **-** | 0 | 0.000 |
| b2965 | speC | **-** | 0.452 | 0.423 |
| b2975 | glcA | **-** | 0.276 | 0.451 |
| b2976 | glcB | **-** | 0.354 | 0.083 |
| b2978 | glcF | **-** | 0.412 | 0.217 |
| b2979 | glcD | **-** | 0.412 | 0.000 |
| b2987 | pitB | **-** | 0.494 | 0.486 |
| b2988 | gsp | **-** | 0 | 0.000 |
| b2994 | hybC | **-** | 0 | 0.000 |
| b2997 | hybO | **-** | 0 | 0.000 |
| b3008 | metC | **-** | 0.51 | 0.510 |
| b3012 | yqhE | **-** | 0 | 0.000 |
| b3018 | plsC | **-** | 1 | 1.000 |
| b3041 | ribB | **+** | 1 | 1.000 |
| b3052 | rfaE | **+** | 1 | 0.000 |
| b3058 | folB | **-** | 1 | 1.000 |
| b3061 | ttdA | **-** | 0 | 0.000 |
| b3062 | ttdB | **-** | 0 | 0.000 |
| b3063 | ygjE | **-** | 0 | 0.000 |
| b3073 | ygjG | **-** | 0 | 0.000 |
| b3089 | sstT | **-** | 0 | 0.000 |
| b3091 | uxaA | **-** | 0 | 0.000 |
| b3092 | uxaC | **-** | 0 | 0.000 |
| b3093 | exuT | **-** | 0 | 0.000 |
| b3111 | tdcGa | **-** | 0 | 0.000 |
| b3112 | tdcGb | **-** | 0 | 0.000 |
| b3114 | tdcE | **-** | 0.138 | 0.474 |
| b3115 | tdcD | **-** | 0.202 | 0.194 |
| b3116 | tdcC | **-** | 0 | 0.008 |
| b3117 | tdcB | **-** | 0.246 | 0.292 |
| b3124 | garK | **-** | 0.002 | 0.016 |
| b3125 | garR | **-** | 0.002 | 0.016 |
| b3126 | garL | **-** | 0 | 0.004 |
| b3127 | garP | **-** | 0 | 0.000 |
| b3128 | garD | **-** | 0 | 0.000 |
| b3132 | agaZ | **-** | 0 | 0.000 |
| b3137 | agaY | **-** | 0 | 0.000 |
| b3161 | mtr | **-** | 0 | 0.000 |
| b3172 | argG | **+** | 1 | 1.000 |
| b3176 | mrsA | **+** | 1 | 1.000 |
| b3177 | folP | **-** | 1 | 1.000 |
| b3187 | ispB | **-** | 0 | 0.000 |
| b3189 | murA | **+** | 1 | 1.000 |
| b3212 | gltB | **-** | 0 | 0.000 |
| b3213 | gltD | **-** | 0 | 0.000 |
| b3222 | nanK | **-** | 0 | 0.000 |
| b3223 | nanE | **-** | 0 | 0.000 |
| b3224 | nanT | **-** | 0 | 0.000 |
| b3225 | nanA | **-** | 0 | 0.000 |
| b3236 | mdh | **-** | 1 | 0.652 |
| b3255 | accB | **-** | 1 | 1.000 |
| b3256 | accC | **-** | 1 | 1.000 |
| b3258 | panF | **-** | 0 | 0.000 |
| b3281 | aroE | **+** | 0.534 | 0.482 |
| b3359 | argD | **+** | 1 | 1.000 |
| b3360 | pabA | **-** | 1 | 1.000 |
| b3365 | nirB | **-** | 0 | 0.000 |
| b3366 | nirD | **-** | 0 | 0.000 |
| b3367 | nirC | **-** | 0 | 0.000 |
| b3368 | cysG | **+** | 0 | 1.000 |
| b3380 | yhfW | **-** | 0.102 | 0.083 |
| b3385 | gph | **-** | 0 | 0.000 |
| b3386 | rpe | **+** | 0.498 | 0.514 |
| b3389 | aroB | **+** | 1 | 1.000 |
| b3390 | aroK | **+** | 0.48 | 0.474 |
| b3403 | pckA | **-** | 0.03 | 0.000 |
| b3409 | feoB | **-** | 0 | 1.000 |
| b3415 | gntT | **-** | 0 | 0.004 |
| b3416 | malQ | **-** | 0 | 0.000 |
| b3417 | malP | **-** | 0 | 0.000 |
| b3425 | glpE | **-** | 0 | 0.004 |
| b3426 | glpD | **-** | 0 | 0.000 |
| b3428 | glgP | **-** | 0 | 0.000 |
| b3429 | glgA | **-** | 1 | 0.000 |
| b3430 | glgC | **-** | 1 | 0.000 |
| b3433 | asd | **+** | 1 | 1.000 |
| b3437 | gntK | **-** | 0 | 0.000 |
| b3450 | ugpC | **-** | 0 | 0.000 |
| b3451 | ugpE | **-** | 0 | 0.000 |
| b3452 | ugpA | **-** | 0 | 0.000 |
| b3453 | ugpB | **-** | 0 | 0.000 |
| b3454 | livF | **-** | 0 | 0.000 |
| b3455 | livG | **-** | 0 | 0.000 |
| b3456 | livM | **-** | 0 | 0.000 |
| b3457 | livH | **-** | 0 | 0.000 |
| b3458 | livK | **-** | 0 | 0.000 |
| b3460 | livJ | **-** | 0 | 0.000 |
| b3493 | pitA | **+** | 0.482 | 0.510 |
| b3500 | gor | **-** | 0 | 0.004 |
| b3517 | gadA | **-** | 0.054 | 0.000 |
| b3519 | treF | **-** | 0 | 0.000 |
| b3526 | kdgK | **-** | 0 | 0.000 |
| b3528 | dctA | **-** | 0 | 0.004 |
| b3551 | bisC | **-** | 0 | 0.008 |
| b3553 | yiaE | **-** | 0.002 | 0.000 |
| b3564 | xylB | **-** | 0 | 0.000 |
| b3565 | xylA | **-** | 0 | 0.000 |
| b3566 | xylF | **-** | 0 | 0.000 |
| b3567 | xylG | **-** | 0 | 0.000 |
| b3568 | xylH | **-** | 0 | 0.000 |
| b3572 | avtA | **-** | 0 | 0.000 |
| b3575 | yiaK | **-** | 0 | 0.000 |
| b3579 | yiaO | **-** | 0 | 0.004 |
| b3581 | sgbH | **-** | 0 | 0.000 |
| b3583 | sgbE | **-** | 0 | 0.000 |
| b3588 | aldB | **-** | 0 | 0.000 |
| b3599 | mtlA | **+** | 0 | 0.000 |
| b3600 | mtlD | **+** | 0 | 0.000 |
| b3603 | lldP | **-** | 0.312 | 0.332 |
| b3605 | lldD | **-** | 0 | 0.000 |
| b3607 | cysE | **+** | 1 | 1.000 |
| b3608 | gpsA | **-** | 1 | 1.000 |
| b3612 | yibO | **-** | 0.32 | 0.304 |
| b3616 | tdh | **-** | 0.012 | 0.004 |
| b3617 | kbl | **-** | 0.012 | 0.004 |
| b3619 | rfaD | **-** | 1 | 0.000 |
| b3620 | rfaF | **-** | 1 | 0.000 |
| b3621 | rfaC | **-** | 1 | 0.000 |
| b3622 | rfaL | **-** | 1 | 0.000 |
| b3626 | rfaJ | **-** | 1 | 0.000 |
| b3627 | rfaI | **-** | 1 | 0.000 |
| b3631 | rfaG | **-** | 1 | 0.000 |
| b3633 | kdtA | **-** | 1 | 0.000 |
| b3634 | coaD | **+** | 1 | 1.000 |
| b3640 | dut | **+** | 0.252 | 0.340 |
| b3642 | pyrE | **+** | 1 | 1.000 |
| b3648 | gmk | **+** | 1 | 1.000 |
| b3653 | gltS | **-** | 0.006 | 0.000 |
| b3654 | yicE | **-** | 0.046 | 0.095 |
| b3665 | yicP | **-** | 0.098 | 0.115 |
| b3666 | uhpT | **-** | 0 | 0.000 |
| b3670 | ilvN | **-** | 0.36 | 0.395 |
| b3671 | ilvB | **-** | 0.36 | 0.395 |
| b3691 | dgoT | **-** | 0 | 0.000 |
| b3692 | dgoA | **-** | 0 | 0.000 |
| b3693 | dgoK | **-** | 0 | 0.004 |
| b3708 | tnaA | **-** | 0.278 | 0.233 |
| b3709 | tnaB | **-** | 0 | 0.000 |
| b3725 | pstB | **-** | 0.024 | 0.004 |
| b3726 | pstA | **-** | 0.024 | 0.004 |
| b3727 | pstC | **-** | 0.024 | 0.004 |
| b3728 | pstS | **-** | 0.024 | 0.004 |
| b3729 | glmS | **+** | 1 | 1.000 |
| b3730 | glmU | **+** | 1 | 1.000 |
| b3731 | atpC | **+** | 1 | 1.000 |
| b3732 | atpD | **+** | 1 | 1.000 |
| b3733 | atpG | **+** | 1 | 1.000 |
| b3734 | atpA | **+** | 1 | 1.000 |
| b3735 | atpH | **+** | 1 | 1.000 |
| b3736 | atpF | **+** | 1 | 1.000 |
| b3737 | atpE | **+** | 1 | 1.000 |
| b3738 | atpB | **+** | 1 | 1.000 |
| b3739 | atpI | **-** | 1 | 1.000 |
| b3744 | asnA | **-** | 0.694 | 0.000 |
| b3748 | rbsD | **-** | 0 | 0.000 |
| b3749 | rbsA | **-** | 0 | 0.000 |
| b3750 | rbsC | **-** | 0 | 0.000 |
| b3751 | rbsB | **-** | 0 | 0.000 |
| b3752 | rbsK | **-** | 0 | 0.000 |
| b3767 | ilvG_1 | **?** | 0.226 | 0.221 |
| b3768 | ilvG_2 | **?** | 0.226 | 0.221 |
| b3769 | ilvM | **-** | 0.226 | 0.221 |
| b3770 | ilvE | **-** | 1 | 1.000 |
| b3771 | ilvD | **+** | 1 | 1.000 |
| b3772 | ilvA | **-** | 0.234 | 0.237 |
| b3774 | ilvC | **+** | 1 | 1.000 |
| b3784 | wecA | **-** | 0 | 0.000 |
| b3786 | wecB | **-** | 0 | 0.000 |
| b3787 | wecC | **-** | 0 | 0.000 |
| b3788 | rffG | **-** | 0 | 0.000 |
| b3789 | rffH | **-** | 0 | 0.000 |
| b3790 | wecD | **-** | 0 | 0.000 |
| b3791 | wecE | **-** | 0 | 0.000 |
| b3793 | wecF | **-** | 0 | 0.000 |
| b3794 | wecG | **-** | 0 | 0.000 |
| b3803 | hemX | **-** | 0 | 0.000 |
| b3804 | hemD | **-** | 0 | 1.000 |
| b3805 | hemC | **+** | 0 | 1.000 |
| b3806 | cyaA | **-** | 0 | 0.000 |
| b3809 | dapF | **+** | 1 | 1.000 |
| b3821 | pldA | **-** | 0.006 | 0.000 |
| b3825 | pldB | **-** | 0.006 | 0.000 |
| b3829 | metE | **+** | 0.51 | 0.538 |
| b3831 | udp | **-** | 0.17 | 0.083 |
| b3833 | ubiE | **-** | 0 | 0.000 |
| b3835 | ubiB | **-** | 0 | 0.000 |
| b3843 | yigC | **-** | 0 | 0.000 |
| b3845 | fadA | **-** | 0 | 0.000 |
| b3846 | fadB | **-** | 0 | 0.004 |
| b3849 | trkH | **-** | 0 | 0.000 |
| b3850 | hemG | **-** | 0 | 1.000 |
| b3870 | glnA | **-** | 0.49 | 0.478 |
| b3892 | fdoI | **-** | 0.06 | 0.008 |
| b3893 | fdoH | **-** | 0.06 | 0.008 |
| b3894 | fdoG | **-** | 0.06 | 0.008 |
| b3902 | rhaD | **-** | 0 | 0.000 |
| b3903 | rhaA | **-** | 0 | 0.000 |
| b3904 | rhaB | **-** | 0 | 0.000 |
| b3907 | rhaT | **-** | 0 | 0.000 |
| b3908 | sodA | **+** | 0 | 0.000 |
| b3909 | kdgT | **-** | 0 | 0.004 |
| b3916 | pfkA | **+** | 0.534 | 0.447 |
| b3917 | sbp | **-** | 0.348 | 0.332 |
| b3918 | cdh | **-** | 0 | 0.000 |
| b3919 | tpiA | **+** | 1 | 1.000 |
| b3926 | glpK | **-** | 0.398 | 0.526 |
| b3927 | glpF | **+** | 0.276 | 0.233 |
| b3929 | menG | **-** | 0 | 0.000 |
| b3930 | menA | **-** | 0 | 0.000 |
| b3939 | metB | **-** | 1 | 1.000 |
| b3940 | metL | **-** | 0.502 | 0.526 |
| b3941 | metF | **+** | 1 | 1.000 |
| b3942 | katG | **-** | 0 | 0.000 |
| b3945 | gldA | **-** | 0.362 | 0.348 |
| b3946 | talC | **-** | 0.102 | 0.162 |
| b3951 | pflD | **-** | 0.054 | 0.162 |
| b3952 | pflC | **-** | 0.054 | 0.162 |
| b3956 | ppc | **-** | 0.482 | 1.000 |
| b3957 | argE | **+** | 1 | 1.000 |
| b3958 | argC | **+** | 1 | 1.000 |
| b3959 | argB | **+** | 1 | 1.000 |
| b3960 | argH | **+** | 1 | 1.000 |
| b3962 | sthA | **-** | 0.032 | 0.051 |
| b3966 | btuB | **-** | 0 | 0.000 |
| b3967 | murI | **+** | 1 | 1.000 |
| b3972 | murB | **+** | 1 | 1.000 |
| b3974 | coaA | **-** | 1 | 1.000 |
| b3990 | thiH | **-** | 0 | 0.170 |
| b3991 | thiG | **-** | 0 | 0.170 |
| b3992 | thiF | **-** | 0 | 0.170 |
| b3993 | thiE | **-** | 0 | 1.000 |
| b3994 | thiC | **-** | 0 | 0.008 |
| b3997 | hemE | **-** | 0 | 1.000 |
| b4005 | purD | **-** | 1 | 1.000 |
| b4006 | purH | **+** | 1 | 1.000 |
| b4013 | metA | **-** | 1 | 1.000 |
| b4014 | aceB | **-** | 0.28 | 0.095 |
| b4015 | aceA | **-** | 0.544 | 0.000 |
| b4019 | metH | **-** | 0.49 | 0.462 |
| b4024 | lysC | **-** | 0 | 0.000 |
| b4025 | pgi | **+** | 0.92 | 0.850 |
| b4031 | xylE | **-** | 0 | 0.000 |
| b4032 | malG | **-** | 0 | 0.000 |
| b4033 | malF | **-** | 0 | 0.000 |
| b4034 | malE | **-** | 0 | 0.000 |
| b4035 | malK | **-** | 0 | 0.000 |
| b4036 | lamB | **-** | 0 | 0.000 |
| b4039 | ubiC | **-** | 0 | 0.000 |
| b4040 | ubiA | **-** | 0 | 0.000 |
| b4041 | plsB | **-** | 1 | 1.000 |
| b4042 | dgkA | **-** | 1 | 0.000 |
| b4053 | alr | **-** | 0.472 | 0.443 |
| b4054 | tyrB | **-** | 0 | 0.000 |
| b4069 | acs | **-** | 0.112 | 0.000 |
| b4077 | gltP | **-** | 0.01 | 0.000 |
| b4079 | fdhF | **-** | 0.338 | 0.028 |
| b4090 | rpiB | **-** | 0.538 | 0.474 |
| b4111 | proP | **-** | 0 | 0.000 |
| b4117 | adiA | **-** | 0.034 | 0.071 |
| b4119 | melA | **-** | 0 | 0.000 |
| b4120 | melB | **-** | 0 | 0.000 |
| b4122 | fumB | **-** | 0.32 | 0.332 |
| b4123 | dcuB | **-** | 0 | 0.000 |
| b4131 | cadA | **-** | 0 | 0.000 |
| b4132 | cadB | **-** | 0 | 0.000 |
| b4138 | dcuA | **-** | 0 | 0.000 |
| b4139 | aspA | **-** | 0.03 | 0.047 |
| b4151 | frdD | **-** | 0 | 0.020 |
| b4152 | frdC | **-** | 0 | 0.020 |
| b4153 | frdB | **-** | 0 | 0.020 |
| b4154 | frdA | **-** | 0 | 0.020 |
| b4160 | psd | **-** | 1 | 0.000 |
| b4177 | purA | **+** | 1 | 1.000 |
| b4196 | sgaH | **-** | 0 | 0.000 |
| b4197 | sgaU | **-** | 0 | 0.000 |
| b4198 | sgaE | **-** | 0 | 0.000 |
| b4208 | cycA | **-** | 0.014 | 0.051 |
| b4226 | ppa | **+** | 1 | 1.000 |
| b4227 | ytfQ | **-** | 0 | 0.000 |
| b4228 | ytfR | **-** | 0 | 0.000 |
| b4229 | ytfS | **-** | 0 | 0.000 |
| b4230 | ytfT | **-** | 0 | 0.000 |
| b4231 | yjfF | **-** | 0 | 0.000 |
| b4232 | fbp | **-** | 0 | 0.000 |
| b4238 | nrdD | **-** | 0.688 | 0.652 |
| b4239 | treC | **-** | 0 | 0.000 |
| b4240 | treB | **-** | 0 | 0.000 |
| b4244 | pyrI | **+** | 1 | 1.000 |
| b4245 | pyrB | **+** | 1 | 1.000 |
| b4254 | argI | **-** | 0.498 | 0.486 |
| b4265 | idnT | **-** | 0 | 0.000 |
| b4266 | idnO | **-** | 0 | 0.000 |
| b4267 | idnD | **-** | 0.032 | 0.028 |
| b4268 | idnK | **-** | 0 | 0.000 |
| b4301 | sgcE | **-** | 0.486 | 0.486 |
| b4321 | gntP | **-** | 0 | 0.000 |
| b4322 | uxuA | **-** | 0 | 0.000 |
| b4323 | uxuB | **-** | 0 | 0.000 |
| b4381 | deoC | **-** | 0.55 | 0.431 |
| b4382 | deoA | **-** | 0.082 | 0.071 |
| b4383 | deoB | **+** | 0.6 | 0.478 |
| b4384 | deoD | **+** | 0.352 | 0.269 |
| b4388 | serB | **-** | 1 | 1.000 |
| b4395 | gpmB | **-** | 0.36 | 0.375 |
| b4407 | thiS | **-** | 0 | 0.170 |

Supplementary data Reference List

Pal,C., Papp,B., Lercher,M.J., Csermely,P., Oliver,S.G., and Hurst,L.D. (2006) Chance and necessity in the evolution of minimal metabolic networks *Nature* **440**: 667-670.
